# Material Requirements of Decent Living Standards

**DOI:** 10.1021/acs.est.3c03957

**Published:** 2023-09-11

**Authors:** Johan Andrés Vélez-Henao, Stefan Pauliuk

**Affiliations:** Faculty of Environment and Natural Resources, University of Freiburg, 8 Tennenbacher Straße 4, 79106 Freiburg, Germany

**Keywords:** decent living standard, material footprint, direct stocks, indirect stocks, life cycle assessment

## Abstract

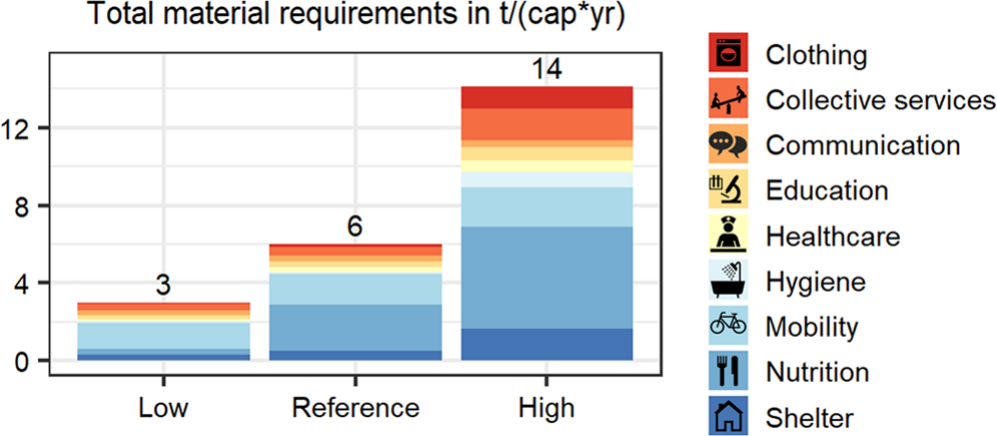

Decent living standards (DLS) provide a framework to estimate a practical threshold for the energy, GHG, and material consumption required to alleviate poverty. Currently, most research has focused on estimating the energy required to provide the DLS. However, no attempt has been made to estimate the material consumption needed to provide the DLS. Thus, we ask the following questions: First, what is the amount of materials in stocks and flows needed to provide a DLS? Second, which lifestyle and technology choices are effective in providing a DLS without creating an excessive demand for additional materials? To provide a DLS, a material footprint (MF) of 6 t/(cap*yr) with a lower and upper bound between 3 and 14 t/(cap*yr) is required. The direct and indirect in-use stocks required are estimated at 32 t/cap and 11 t/cap, respectively. Nutrition (39%) and mobility (26%) contribute the most to total MF. Buildings account for 98% of direct stocks, while the construction sector accounts for 61% of indirect stocks. We extend the coverage of the DLS by including the collective service dimension and link the material stock-flow-service nexus and life cycle assessment to compute the MF and in-use stocks needed to provide the DLS.

## Abstract 2

Little research has been devoted to understanding the nexus between decent living standards (DLS) and material consumption. We estimate for the first time the material flows and stocks needed to provide the DLS.

## 1 Introduction

### 1.1 Motivation

The recent report on the progress of sustainable development goals (SDGs) published by the United Nations (UN)^1^ showed that the trends in eradicating poverty and hunger, reducing inequalities, and increasing access to basic needs have stalled or reversed.^1,2^ Therefore, immediate action is needed on a global scale to put the world on the path to sustainability and well-being for all.^1^

However, there is no consensus on the basic access levels for a number of specific services, including public buildings and infrastructure, clothing, or nutrition that enable well-being.^3^ This is in part because sociocultural factors and individual circumstances shape the notion of well-being.

To overcome this gap and to indicate universal determinants of human well-being, Rao & Min^4^ suggest a bundle of services, independent of sociocultural factors, to be essential preconditions to human well-being. These services can be interpreted as the minimum requirements that enable a decent living standard (DLS). The DLS offers a simple but efficient bottom-up approach to understand the tradeoffs between poverty and climate change, as well as a framework to estimate the demand for energy and materials required to achieve the SDGs (e.g., no poverty (SDG 1), zero hunger (SDG 2), good health and well-being (SDG 3), quality education (SDG 4), and reduced inequalities (SDG 10)).^5^

In this respect, DLS research has focused on estimating the energy necessary to supply the DLS in various social–cultural contexts. Focusing on the global South (India, Brazil, and South Africa), Rao et al.^6^ estimate that by 2050, the energy demand necessary to provide a DLS is in the range of 10.1–20.9 GJ/(cap*yr). More broadly, Millward-Hopkins et al.^7^ estimate that the global average energy requirements to fulfill a DLS by 2050 is around 15.3 GJ/(cap*yr). Alternatively, Kikstra et al.^8^ estimate that by 2040 at the regional level, between 9 and 36 GJ/(capita*yr) of energy is needed to fill the gap between current multidimensional poverty and the DLS.

These findings provide valuable information about the DLS in low-energy demand scenarios in line with temperature targets of 1.5–2 °C; however, they do not address the link between DLS and other environmental impact categories, such as material consumption. The link to materials, in particular, is relevant because materials such as steel, wood, and concrete form an important link between poverty-related and environmental SDGs. On the one hand, poverty eradication requires access to basic services, which require functioning products and material stocks for their provision. On the other hand, material production is a major contributor to greenhouse gas (GHG) emissions (23% of global GHG in 2015)^9^ and is responsible for more than 90% of biodiversity loss and water stress.^10^ Additionally, the consumption of materials is increasing at unsustainable rates (23-fold throughout the 20th century).^9,11^ A trend that likely will continue to grow as the transition to clean energies and more efficient products requires a massive amount of materials; e.g., photovoltaic and wind power plants require between 6 and 40 times more copper than conventional fossil fuel technologies.^12^ These material–environment links are the foundation of an ongoing debate on the need for establishing a cap on material consumption.^13^

### 1.2 Objectives

This paper aims to answer two questions: What is the amount of materials, in-use stocks, and extraction flows needed to provide a DLS and which lifestyles and bundles of efficiency strategies are effective in providing a DLS without creating excessive demand for additional resources?

To answer these questions, we made two method improvements. First, we extend the coverage of the DLS by including the collective service dimension by including the building space associated with wholesale and retail, offices, hotels and restaurants, sports facilities, and others. Second, we provide a link between the material stock-flow-services nexus^14^ and ecoinvent, a widely used database for LCA analysis, to compute the material footprint (MF), in-use stocks, and industrial material stocks needed to deliver the DLS, previously unseen in the literature.

Outside of our scope is the regionalization of the DLS and the effect of efficiency strategies and policy targets, i.e., rebound effects on the provisioning of the DLS mainly because such analysis requires additional data and assumptions.

## 2 Methods

### 2.1 Background

This study integrates the Rao & Min^4^ DLS framework and the quantitative energy thresholds for each DLS dimension from Millward-Hopkins et al.^7^ with the stock-flow-service nexus^14^ and the energy service cascade concepts,^15^ both key concepts to link material flow analysis to social and environmental sustainability aspects.

### 2.2 Analytical Method

**Figure 1:**
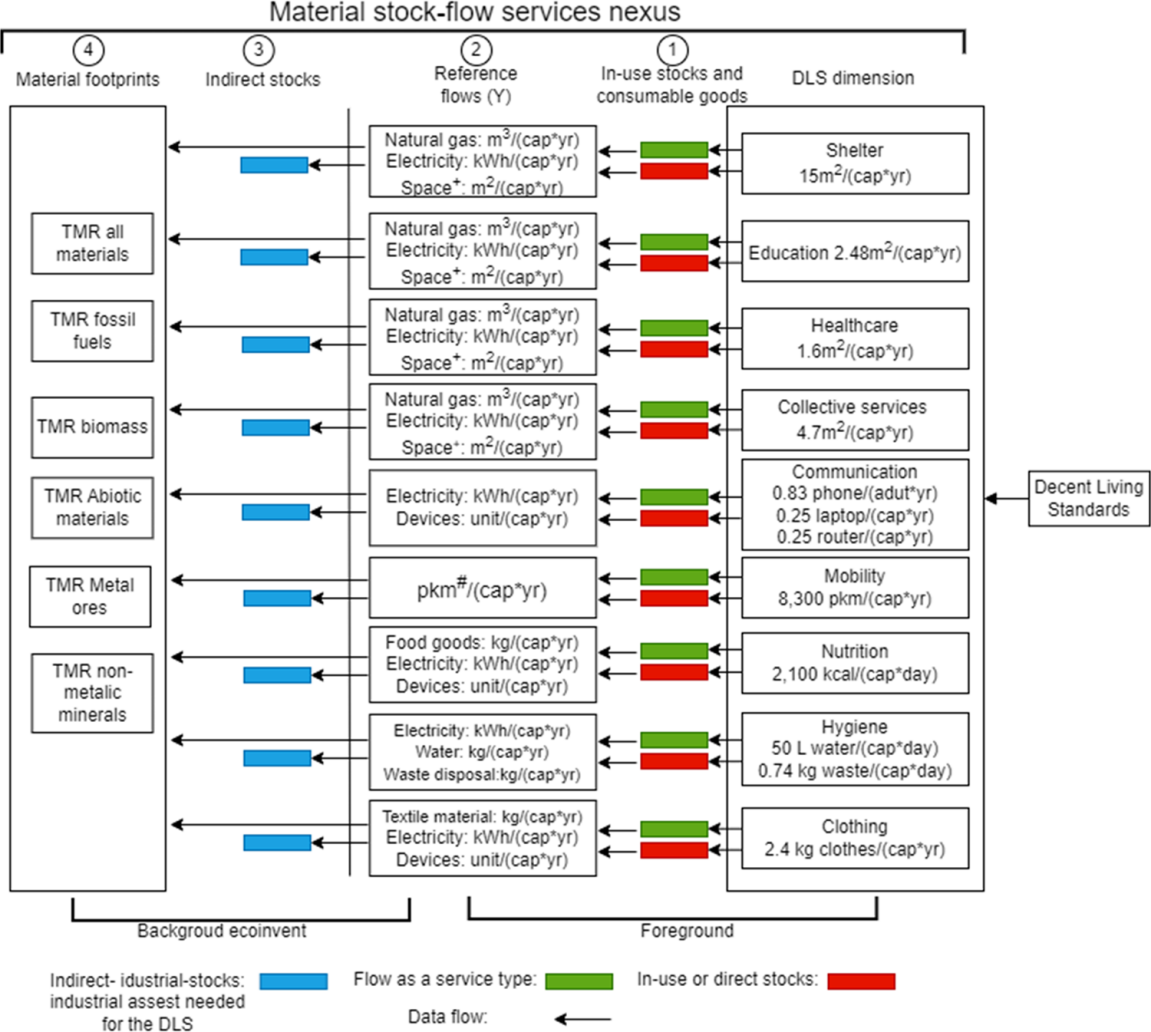
Conceptual framework of the material stock-flow-service nexus for the decent living standards (DLS). DLS levels are based on Millward-Hopkins et al.^7^ Total material requirement (TMR). Capita (cap). Passenger-kilometer (pkm). #: in ecoinvent, the transport services are provided in pkm or vehicle–km, and this service description accounts for the operational energy, the vehicle production (downscaled), and the infrastructure (e.g., roads, downscaled) needed to provide the service. This information is extracted from the database and recorded as in-use stocks. +: data from the RECC model are used to translate the space in m^2^ into kg of building materials to be ready to use in ecoinvent. A complete list of the reference flows by DLS dimension is provided in Supporting Information SI2.

The method to estimate the DLS MF consists of four steps. First, we compile a list of services required by each DLS dimension and link them to their respective provisioning systems as seen in Figure 1. Two types of provisioning system are considered: flow as a service, which represents flows such as food consumption for services such as nutrition, and stock-operation type, where an in-use stock, such as a building, vehicle, IT device, or household appliance, is operated to provide a service to end users.

In the second step, the reference flows for the different provisioning systems are calculated from the stock-flow-service nexus.^14^ We compile a bundle of services and associated in-use stocks needed to provide a DLS in each dimension based on Rao & Min^4^ and Millward-Hopkins et al.^7^ We use the global average population for the year 2023 from the UN^7^ as a reference for our analysis. For shelter, we assume a four-person household with a space floor of 15 m^2^/cap. Similarly, for education, healthcare, and collective services, a space floor of 2.48, 1.6 , and 4.7 m^2^/cap, respectively, was considered. For communication, each person over the age of 10 yrs requires a phone, while one laptop and one router are required per household. For mobility, approximately 8300 passengers-kilometers (pkm) are assumed. For nutrition, about 2100 kcal/(day*cap) is required. Additionally, for hygiene, 50 L/(day*cap) of water is required and 0.74 kg of waste/(cap*day) is generated. Finally, for clothing, 2.4 kg of clothes/(cap*yr) are required (see Figure 1; detailed information on the main assumption and estimation of each reference flows by DLS dimension is provided in Supporting Information SI1).

In the third step, the indirect stocks in the form of industrial capital (for example, material stocks in the steel industry that are attributed to transportation services through the steel required for the vehicles) are estimated. Finally, the reference flows are linked to their respective environmental flows.

As each DLS dimension can be fulfilled by several sets of goods and services, we perform a scenario analysis to account for individual choices and technological options. For example, in the case of nutrition, a daily intake of 2,100 kcal/cap can be met by different diets, e.g., carnivorous, vegetarian, or vegan. These differences in lifestyles lead to differences in MF. Thus, we construct a total of 6441 different scenarios (constituted of 3456 scenarios for nutrition, 384 for shelter, 288 for hygiene, 320 for clothing, 9 for communication, 960 for mobility, 512 for health, 256 for education, and 256 for collective services). Moreover, we conduct 5 additional scenarios to understand what effect has different electricity grids (3 grids in which hydro, wind, and nuclear are the main energy source, one in which solar has high shares and one in which high shares of renewables but not nuclear are used) in providing a DLS (see SI1 and SI2).

### 2.3 Process-Based Life Cycle Assessment (P-LCA)

To estimate the DLS MF, we use a process-based life cycle assessment (P-LCA) and the ecoinvent 3.8 database. Particularly, we utilize the system model “allocation, cut-off by classification” which means the environmental burdens are the responsibility of the producers and are not allocated to recyclable products, therefore incentivizing the use of recyclable products.^16^ As our analysis is based on a global average DLS, For consistency from ecoinvent, we take the unit processes accounting for the world-average technology (i.e., processes with global or rest-of-the-world geographical scope).

P-LCA has two desired features that make it a more suitable approach to estimate the MF of providing a DLS compared to other methods such as environmentally extended input–output analysis (EEIO) (see Giljum et al.^17,18^ for an overview). First, P-LCA has a greater product and service resolution than EEIO models, allowing us to account for the bundles of flows and in-use stocks needed to provide the DLS. In other words, with P-LCA, it is possible to distinguish between the different types of technologies within a particular sector, such as between electric (EV) and combustion vehicles (ICEV), and between residential and nonresidential buildings, while in most EEIO models, industries are represented by one aggregated product (e.g., the transport sector aggregates EV and ICEV and many others transport modes in one). Second, the products and services required to fulfill a certain need, such as shelter or transport, are provided in physical units, e.g., m^2^ or pkm, while in EEIO data are presented in monetary units. The former is more directly related to lifestyles and daily needs and activities. These two properties allow us to estimate the DLS MF with a high level of technological detail.

Data for buildings (residential and non-residential) are taken from the RECC database.^19^ This is because, in ecoinvent, such building types are poorly represented. The RECC data contain information on the main construction materials (e.g., concrete, steel, wood, paper, and cardboard) and energy consumption for several building archetypes and regions (see SI1).

The MFs were calculated using the characterization factors (CFs) (the impact intensity of a substance relative to a common reference), for the total material requirement (TMR, total extracted material) and the raw material input (RMI, total processed materials, equals the TMR without harvest residues and overburden) provided by Pauliuk.^20^ We consider the two impacts, as both are used in different contexts. We provide results and discussion for the TMR (hereafter MF), while the results for RMI without discussion are presented in SI3-4 as ancillary data.

## 3 Results

The findings represent the provisioning of a DLS with a global average coverage for a single person in the hypothetical case of absolute or abject poverty, i.e., zero or scarce access to any provisioning services. The flows and in-use stocks are estimated based on the thresholds for the DLS presented in Figure 1. Table 1 presents a summary of the four material indicators by DLS dimension.

**Table 1:** Summary of Material Indicators for Decent Living Standards

DLS dimension	service provision	reference flow (unit/(cap*yr))^c^	direct stocks (kg/cap)	indirect stocks (kg/cap)	material footprint (kg/(cap*yr))
shelter: 15 m^2^/(cap*yr)	cement, portland	8 kg/(cap*yr)	604^b^	234^d^	16^e^
	paper	0.01 kg/(cap*yr)	1^b^		0.1^e^
	wood	0.02 m^3^/(cap*yr)	776^b^		40^e^
	concrete	0.1 m^3^/(cap*yr)	18,462^b^		261^e^
	steel	13 kg/(cap*yr)	1067^b^		95^e^
	natural gas	40 m^3^/(cap*yr)			40
	electricity	101 kWh/(cap*yr)			69
	lamps	1 unit/(cap*yr)	0.09		9^e^
nutrition: 2084 kcal/(cap*day)	red meat	22 kg/(cap*yr)		4539^d^	246
	chicken	34.5 kg/(cap*yr)			230
	milk	179 kg/(cap*yr)			56
	tomatoes	340 kg/(cap*yr)			376
	sugar	31 kg/(cap*yr)			220
	vegetable oil	30 kg/(cap*yr)			306
	lentils	11 kg/(cap*yr)			10
	potatoes	270 kg/(cap*yr)			323
	electricity	30 kWh/(cap*yr)			20
	natural gas	39 m^3^/(cap*yr)			40
	refrigerator	0.03 unit/(cap*yr)	14.5		18^e^
	stove	0.02 unit/(cap*yr)	14.5		10^e^
clothing: 2.4 kg clothes/(cap*yr)	electricity	82 kWh/(cap*yr)		318^d^	56
	washing machine	0.05 unit/(cap*yr)	38		49^e^
	woven cotton	0.4 kg/(cap*yr)			7
	knit cotton	0.4 kg/(cap*yr)			6
	synthetic rubber	0.6 kg/(cap*yr)			3
	wool	1 kg/(cap*yr)			40
hygiene: 50 L water/(cap*day) 0.74 kg waste/(cap*day)	natural gas	32 m^3^/(cap*yr)		1109^d^	33
	tap water	18,250 kg/(cap*yr)			26
	waste treatment, open dump	–89 kg/(cap*yr)^a^			
	waste treatment, open burning	–30 kg/(cap*yr)^a^			
	waste treatment, unsanitary landfill	–68 kg/(cap*yr)^a^			0.17
	waste treatment, biowaste	–14 kg/(cap*yr)^a^			0.07
	waste treatment, sanitary landfill	–70 kg/(cap*yr)^a^			13
	residential wastewater treatment	–18 m^3^/(cap*yr)^a^			38
education: 2.48 m^2^/(cap*yr)	wood	0.01 m^3^/(cap*yr)	402^b^	350^d^	21^e^
	concrete	0.01 m^3^/(cap*yr)	1972^b^		28^e^
	steel	1 kg/(cap*yr)	109^b^		10^e^
	bricks	3 kg/(cap*yr)	206^b^		9^e^
	natural gas	8.4 m^3^/(cap*yr)			9
	electricity	263 kWh/(cap*yr)			179
	lamps	4 unit/(cap*yr)	0.35		37^e^
healthcare: 1.6 m^2^/(cap*yr)	wood	0.01 m^3^/(cap*yr)	324^b^	208^d^	17^e^
	concrete	0.01 m^3^/(cap*yr)	1211^b^		17^e^
	steel	0.91 kg/(cap*yr)	73^b^		6^e^
	bricks	3 kg/(cap*yr)	211^b^		9^e^
	natural gas	5 m^3^/(cap*yr)			6
	electricity	240 kWh/(cap*yr)			163
	lamps	4 unit/(cap*yr)	0.35		37^e^
communication: 0.83 phone/(adult*yr) 0.25 laptop/(cap*yr) 0.25 router/(cap*yr)	electricity	364 kWh/(cap*yr)		335^d^	248
	smartphone	0.33 unit/(cap*yr)	0.06		16^e^
	laptop	0.06 unit/(cap*yr)	0.36		18^e^
	internet (router)	0.04 unit/(cap*yr)	0.45		12^e^
mobility: 8274 pkm/(cap*yr)	bicycle	1460 person*km	16	3365^d^	43
	motor scooter	841 person*km	8		86
	passenger car	2806 km^c^	599		1,149
	regular bus	1083 person*km	10		143
	train	433 person*km	2		55
	aircraft	1650 person*km	0.15		80
collective services: 4.7 m^2^/(cap*yr)	wood	0.02 m^3^/(cap*yr)	762^b^	335^d^	40^e^
	concrete	0.02 m^3^/(cap*yr)	4162^b^		59^e^
	steel	3 kg/(cap*yr)	204^b^		18^e^
	bricks	5 kg/(cap*yr)	377^b^		16^e^
	natural gas	18 m^3^/(cap*yr)			18
	electricity	392 kWh/(cap*yr)			266
	lamps	4 unit/(cap*yr)	0.35		37^e^
total			32,000	11,000	6000

a Waste treatment processes in ecoinvent are given in negative values.

b Direct stock accounting for buildings by the amount of materials needed to deliver a certain space in m^2^.

c Passenger car in ecoinvent is given in km.

d Indirect stocks represent the result of each DLS dimension.

e Indicates the material footprint for stocks, while no symbol represents the footprint for flows. Details are given in SI3-4.

### 3.1 Material Footprint

**Figure 2:**
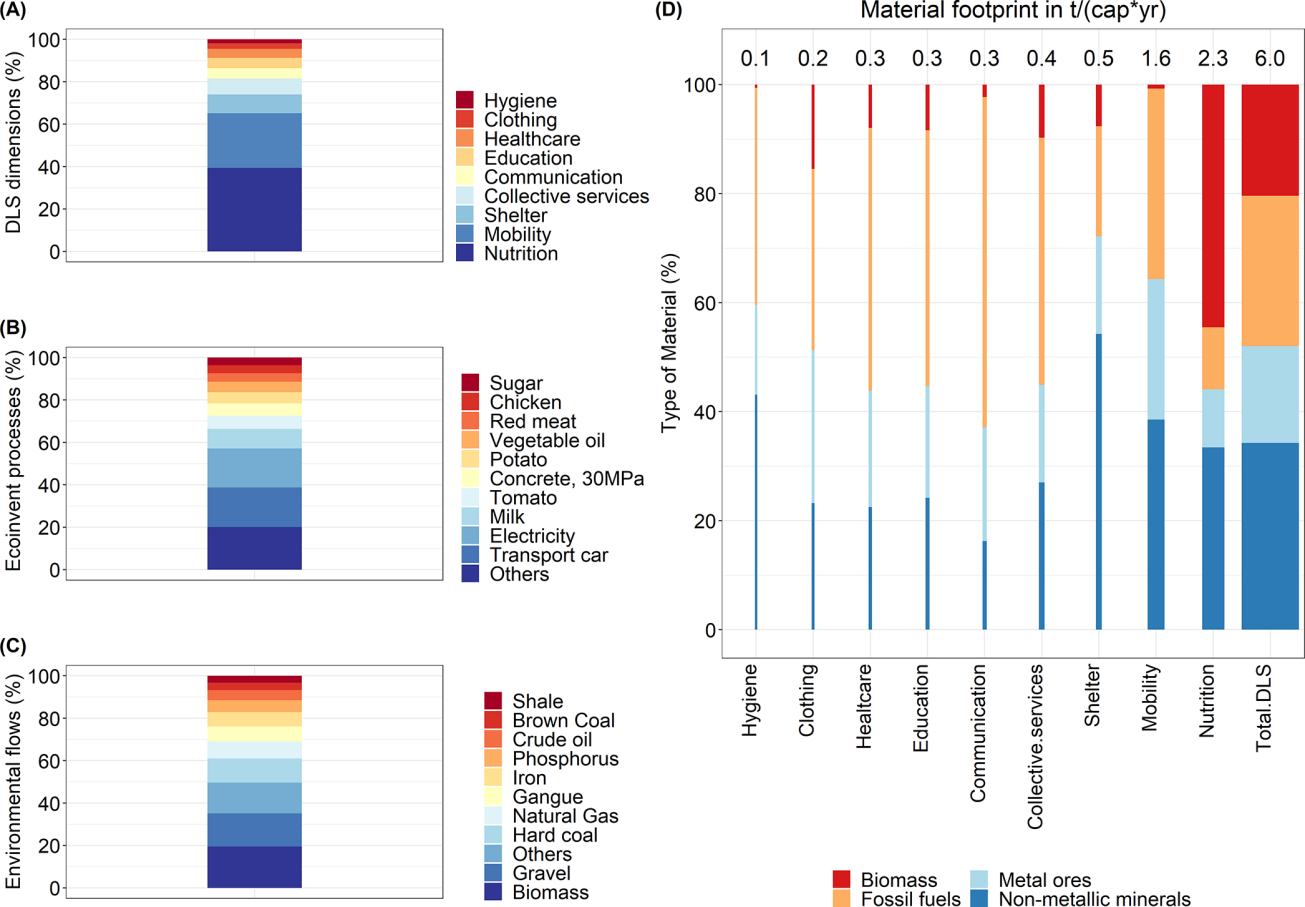
Four perspectives on the total material requirements (TMR) of the decent living standard (DLS) by DLS dimension (A), by provisioning services (B), by the top 10 environmental flows (C), and by DLS dimension and the type of material (D). The TMR for (A–C) is 6 t/(cap*yr) matching values from (D). The width of the (D) bars is proportional to the TMR. Data for panels (A–D) are provided in Supporting Information SI3. The results shown in panels (A) and (B) are calculated using equation 7 and panels (C) and (D) using equation 8 in Supporting Information SI1. The ecoinvent unit processes in panel (B) are the flows listed in Figure 1 (step 2).

Providing a DLS requires an MF of about 6 t/(cap*yr) of which the dimension of nutrition (38%) and mobility (26%) are the categories that contribute the most to the total (see Figure 2A). The high contribution of nutrition is mainly due to the fact that this dimension is associated with high amounts of biomass (1 t/(cap*yr)) and non-metallic minerals (0.8 t/(cap*yr)), the latter mainly related to the use of pesticides and fertilizers.

Looking at services, transport by car (19%) and electricity (17%) are the most significant contributors to the total MF, while food products contribute with around 37% of the total footprint, milk provision being the most significant contributor (see Figure 2B). Compared to other food products, milk has a significant contribution due to two factors: the amount of milk (∼0.49 gr/cap) needed to meet the daily requirements and the MF associated with the milk (3.15kg/kg). For example, the amount of tomatoes required to meet the daily diet (∼0.9 gr/cap) is higher than the milk but has a lower MF (1.10 kg/kg); on the other hand, the red meat (∼0.1 gr/cap) needed to meet the daily diet is less than the milk, but it has a higher MF (10.8 kg/kg).

The environmental flows (that is, flows linked to the footprint of CFs) that most contribute to the MF are biomass (20%), gravel (16%), and hard coal (11%), while 14% of the total footprint comes from metal ores such as gangue and iron. Similarly, phosphorus and shale (non-metallic minerals) contribute with 9% of the total MF (see Figure 2C). In addition, 85% of the environmental flows associated with biomass are related to the nutrition dimension, while 50 and 18% of the flows of gravel and hard coal come from the dimension of mobility and education, respectively (see Figure 2B).

When looking at the types of materials, non-metallic minerals (such as sand, gravel, limestone, and clay) and fossil fuels are responsible for around 34 and 28% of the total footprint, respectively, while metal ores contribute with 18% of the total impacts (see Figure 2D).

Collective services have an MF of 0.4 t/(cap*yr). In this dimension, electricity and gas consumption represent 59 and 4% of the total impacts, respectively, while 8 and 29% of the impacts are attributed to the lamps needed for illumination and the materials that make up the building (bricks (4%), wood (9%), steel (4%), and concrete (13%)), respectively (see SI3.10). Similarly, by the type of MF, the fossil fuels (45%) contribute with the most impact, while the non-metallic minerals, metal ores, and biomass contribute with the remaining 27, 18, and 10%, respectively (see SI3.21).

The DLS dimension that contributes less to the MF is communication (0.1 t/(cap*yr)) in this dimension; electricity consumption (364 kWh/(cap*yr)) accounts for 83% of the total impacts. This is mainly due to the use of Wi-Fi and networks (212 and 355 kWh/year) (see SI1).

### 3.2 Direct and Indirect Material Stocks

**Figure 3:**
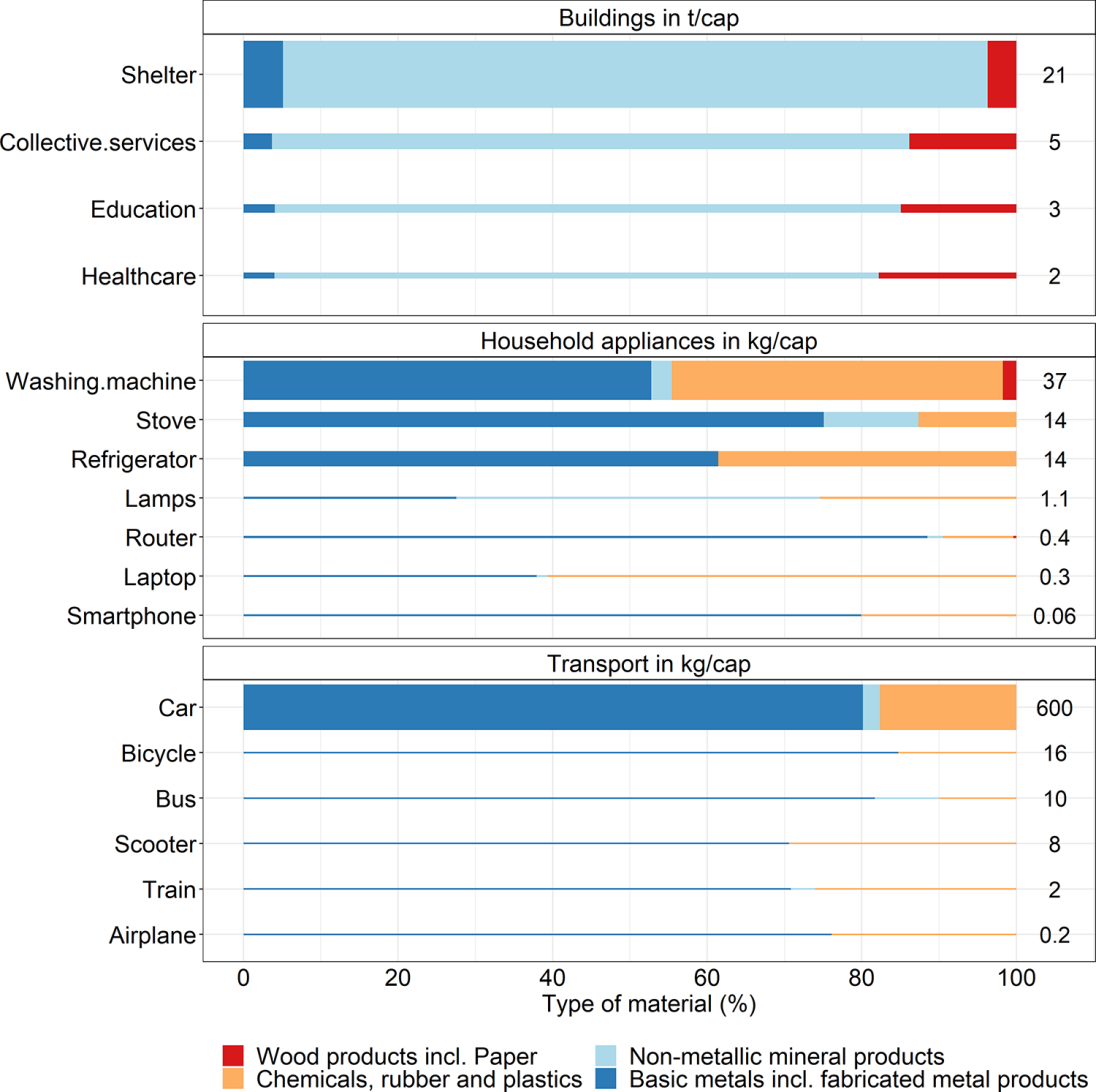
Direct stock associated with a decent living standard. For visualization purposes, the values are rounded. The width of each bar is proportional to the value of the highest stock in each category. Wood and paper are aggregated into one category. Chemicals, rubber, and plastics were merged into one category, and basic metals and fabricated metal products were aggregated in Basic metals incl. fabricated metal products. For transport, direct stocks include vehicles, whereas transport infrastructure such as roads is part of indirect stocks. The values in the figure are the direct stocks shown in Table 1. Data for the figure are provided in Supporting Information SI4.1. The results shown in the figure are calculated with equation 13 in Supporting Information SI1.

The direct stocks required to provide the DLS is ∼32 t/cap, with 98% of the direct stocks associated with the building. Precisely, shelter, education, healthcare, and collective services represent 66, 9, 6, and 17% of the total direct stocks, respectively (see Figure 3). Moreover, between 78% (healthcare) and 91% (shelter) of the materials of the building stock are non-metallic materials, whereas wood materials account for between 4% (shelter) and 18% (healthcare). For the collective services, 82% of the total stocks are made up of non-metallic minerals, while wood and paper products, basic metals, and chemicals and rubber contribute with 14, 4, and <1% of the remaining total respectively.

The material stocks of the household appliances are mainly composed of basic metals and chemicals, rubber, and plastics. The former represents between 28% (lamp) and 89% (router) of the total materials. The latter represents between 9% (router) and 61% (laptop) of the total materials. Similarly, the material composition of the transport stocks is mainly basic metals (between 71% for scooter and 85% for bicycle) and chemicals, rubber, and plastics (between 10% for bus and 29% for scooter).

**Figure 4:**
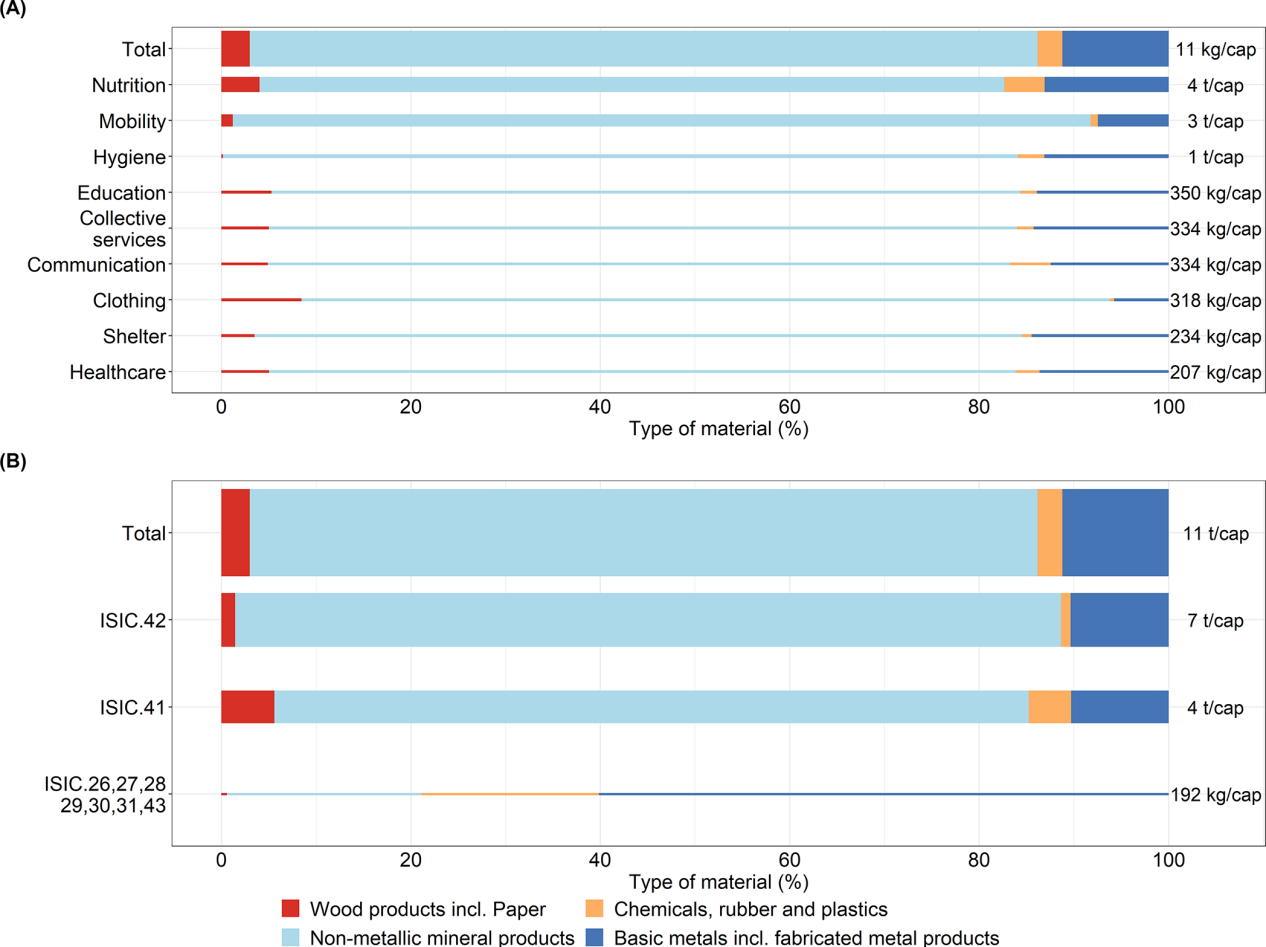
Two perspectives of the indirect stocks required to provide a decent living standard (DLS) by dimension (A) and by capital goods according to the international standard classification of economic activities (ISIC) (B). The width of each bar is proportional to the value of the total indirect stocks. ISIC 26 Manufacture of computer, electronic and optical products, ISIC 27 Manufacture of electrical equipment, ISIC 28, manufacture of machinery and equipment; ISIC 29, manufacture of motor vehicles, trailers, and semitrailers; ISIC 30, manufacture of other transport equipment; ISIC 31, manufacture of furniture; ISIC 41, construction of buildings; ISIC 42, civil engineering; and ISIC 43, specialized construction activities. The values in panel (A) are the indirect stock shown in Table 1. Data for panels (A) and (B) are provided in SI4.19–39. The results shown in the figure are calculated with equation 11 in SI1.

The indirect material stocks in industrial assets associated with DLS are ∼11 t/cap (see Figure 4A). The dimensions of nutrition (42%) and mobility (31%) contribute the most to indirect stocks. In both dimensions, the non-metallic mineral and basic metal products jointly account for 92% (nutrition) and 98% (mobility) of the total materials. A similar trend can be observed for the remaining DLS dimensions.

The collective service dimension contributes with around 3% of the total indirect stocks. In this category, non-metallic mineral products account for 79% of the total impacts, while basic metals, wood products, and chemicals and rubber contribute with the remaining 14, 5, and 2%, respectively.

Looking at the types of indirect stocks, it can be seen that 61% arise from the construction sector, in particular civil engineering projects (ISIC 42) such as roads, railways, tunnels, and bridges, utility projects (e.g., pipelines, irrigation systems, reservoirs, and power plants), and engineering projects (e.g., refineries, chemical plants, waterways, dams, and outdoor sports facilities). Furthermore, the remaining 37 and 2% of indirect stocks come from the construction of buildings (ISIC 41) (residential and nonresidential buildings such as factories, hospitals, hotels, airports, parking garages, warehouses, and religious buildings) and other sectors (ISIC 26 to 31 and 43) such as the manufacture of machinery and equipment and others, respectively (see Figure 4B). In general, non-metallic mineral products and basic metals account for 94% of the total indirect stocks.

### 3.3 Scenario Analysis

**Figure 5:**
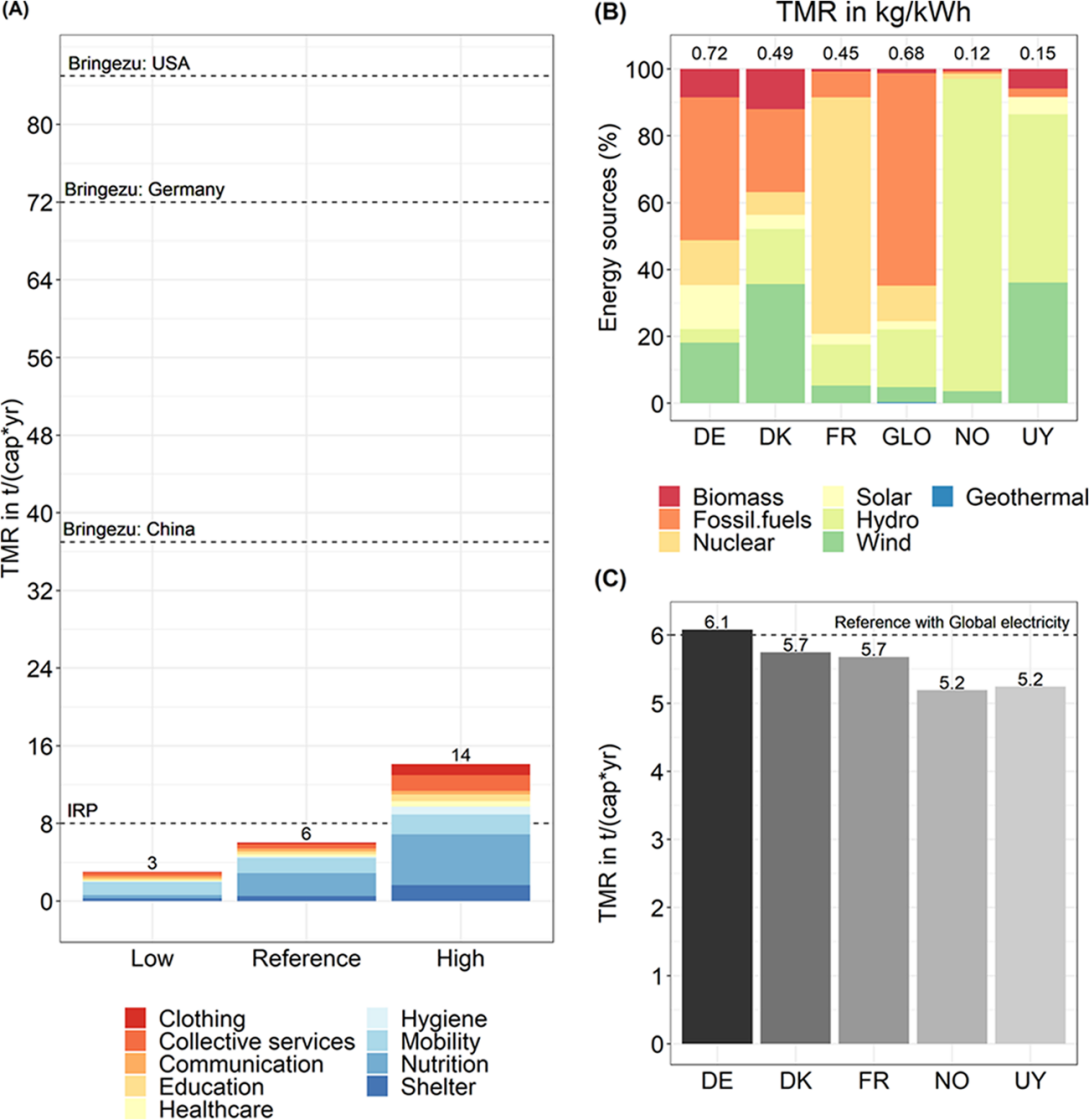
Scenario results for the total material requirement (TMR) footprint for the decent living standard (DLS), showing lower and upper bond scenario results (A), TMR by selected electricity mix in kg/kWh (B), and TMR for DLS based on the reference scenario with different electricity mixes (C). Dashed line data in panel (A) represent the material cap target from IRP^21^ and the current TMR for the selected countries from Bringezu.^22^ Dashed line data in panel (C) represent the TMR of the reference scenario (6 t/(cap*yr)). Data for the plots are provided in SI2.

Lifestyles and efficiency measures significantly influence the MF. The findings suggest that the DLS can be provided with an MF in the range of 3–14 t/(cap*yr) (see Figure 5A).

A DLS with an MF of 3 t/(cap*yr) can be achieved through a multifamily residential household constructed with wood and little concrete and steel (shelter), a vegan diet with potatoes as a staple (nutrition), non-residential buildings with more wood (education, healthcare, and collective services), and a scenario in which the use of private cars is reduced by 27% and short distances are covered by walking (mobility) (B2DS scenario, see SI1). Alternatively, a DLS with an MF of 14 t/(cap*yr) can be achieved through a residential tower building (shelter), a diet based on meat and rice as a staple (nutrition), standard buildings (education, healthcare, and collective services), and the provision of mobility by electric vehicles (see SI1 and SI5).

The energy scenarios show that it is possible to lower the MF for providing a DLS (up to ∼1.0 t) if electricity is provided with renewable energies. Having a grid with high shares of wind (36%) (Denmark) may reduce the MF by 5%. Alternatively, an electricity grid with hydro as the main energy source (93%) (Norway) may bring 14% savings. The highest amount of nuclear power (71%) (France) translates into 6% of the savings. The high shares of renewable sources without nuclear sources (Uruguay) translate into 13% of the potential savings in providing a DLS. However, a mix of high shares of fossil fuels (43%) and solar resources (13%) (German) will increase the MF by 1%. This is mainly because although the German mix has more solar (13%) and wind (18%) sources than the global mix (reference scenario 2 and 4%, respectively), the former has less hydro (4%) than the global mix (17%).

## 4 Discussion

### 4.1 DLS MF and Material Decoupling Targets

The MF (3–14 t/(cap*yr)) to provide a DLS is in line with the suggestions for a sustainable consumption of materials by 2050 found in the literature. Specifically, the International Resource Panel (IRP) suggests that an MF of 6–8 t/(cap*yr) is required to decouple economic growth from the use of natural resources.^21^ Similarly, Bringezu^22^ suggests that a potential target for achieving sustainable consumption of natural resources is an MF in the range of 8–14 t/(cap*yr). Additionally, Lettenmeier et al.^23^ suggest a material cap of 8 t/(cap*yr) in Finland to ensure sustainable consumption of materials in the household sector.

Compared with current material consumption patterns, the DLS is much lower. For example, MFs associated with consumption in the US, Germany, and China are around 85, 72, and 37 t/(cap*yr), respectively (see Figure 5A, dashed lines and Bringezu et al.^24^ for additional MF for different EU countries for a timespan between 1993 and 2000). Furthermore, Wiedmann et al.^100^ provided information for the RMI for 186 nations for the year 2008. These results cannot be compared with the values presented in Figure 5A for TMR but with the Supporting results in SI3.

Finally, our results represent the long-term average MF for maintaining stocks with an average lifetime (80 yrs for buildings) and not the actual stock dynamics and associated impacts that have a different development over time than the service consumption. Additionally, individual countries may have a much longer lifetime for stocks than what we have used here, while other countries recently have expanded (e.g., China) and close-to-saturated (e.g., Germany, Japan) stocks so that there are lower maintenance and replacement requirements in the coming decades, leading to lower MFs even for high living standards.^25^

### 4.2 Role of In-Use Stocks and Energy Provisioning in the DLS MF

Buildings make up a large proportion of direct stocks (98% of the total) since their construction is highly material-intensive and has a longer lifespan (≥80 yrs) than other stocks, such as cars (∼15 yrs). Therefore, alternative construction processes such as changing the materials or making the construction lighter are necessary to provide a DLS with low material intensity.^25^ Taking the reference scenario for the shelter (a single-family building) as an example, increasing the amount of wood (from ∼52 to ∼120 kg/m^2^ or 131%) would reduce the MF by 26%. Similarly, a lighter building using less concrete (from ∼1.2 ton/m^2^ to ∼886 kg/m^2^ or 28%) and steel (from ∼71 to ∼55 kg/m^2^ or 22%) would reduce the MF by 17%. Combining the two alternatives (more wood and less concrete and steel) will reduce the MF by 28%. Alternatively, switching to a multifamily household (standard building types) reduces the MF by 25%, while changing to a residential tower will increase the MF by 67%. Finally, moving to a lighter, wood-based multifamily building would reduce the MF by 43%. Similar trends can be observed for nonresidential buildings (see SI5.2).

In general, the total stocks (direct and indirect) required to provide a DLS (∼42 t/cap) are significantly lower than those reported for developed countries (335 t/cap), China (136 t/cap) and the world average (115 t/cap), while for developing countries (38 t/cap), our results are slightly higher.^11^ This implies that on average, in developing countries, stocks need to build up to provide a DLS. Results from Krausmann et al.^11^ differ methodologically from ours, as the former are based on historical data of accumulated stocks across the years (material flow analysis), and ours are based on a product system model, where, to provide any particular service, stocks are built up from zero (LCA perspective).

The mix of electricity chosen to provide a DLS has direct implications for the MF (1.0 t/(cap*yr) or 17% of total impacts). We use a global average technology mix with a share of 63% fossil fuels, 17% hydro, 11% nuclear, 4% wind, and 5% others (geothermal, biogas, biomass, solar, and waste).^26^ This result may be lower if electricity grids with high amounts of renewable energy are used. For example, using the mix from Norway that is mainly based on hydro (93% of the total shares) reduces the MF for providing a DLS to 5.2 t/(cap*yr) or 14%. Alternatively, using the German mix with fewer fossil fuels (43% of the total shares) and hydro (4% of the total shares) but more solar (13% of the total shares) compared to the global average technology used will slightly increase the DLS MF by 1% (see Figure 5C).

In general, electricity mixes with high shares of renewables (especially hydro) help to provide a DLS with a lower MF, in contrast to what one might expect due to the fossil fuel–metal ore linkage.

### 4.3 Impacts of Lifestyles and Technology Choices on the DLS MF

Different lifestyles and efficiency measures significantly change the MF of providing a DLS. Taking nutrition as an example, reducing meat consumption by half or switching to a vegetarian or vegan diet, leaving all of the other parameters constant, reduces the MF of nutrition by 9, 24, and 35%, respectively. Alternatively, switching the consumption of staples to wheat or rice (potato reference scenario), assuming that the consumption in gr/day is the same regardless of the type of staple, will increase the MF between 22 and 34% respectively, mainly due to two factors. First, the yields for potatoes (21 t/ha) are between 4- and 5-fold bigger than for rice or wheat, respectively,^27^ and second, the amount of fertilizer (nitrogen N, phosphorus P, and potassium K) used per kg of potatoes (∼0.04 kg fertilizers/kg) is lower than that required for rice (∼0.06 kg fertilizers/kg) or wheat (∼0.08 fertilizers/kg) (own estimations based on fertilizer recommendations from FAO^27,28^). Furthermore, switching to more efficient cooking appliances (80% compared to reference) will reduce the MF by 2%, while cooking with electricity instead of gas will increase the MF by 10%.

Assuming that by 2030 the regions will adjust their daily diet to meet the healthy global diets (HGD) recommendations^29^ and other parameters such as energy provision and consumption remain similar, the MF for the nutrition dimension in regions where rice is consumed as a staple, such as Asia (77.5 kg/cap), will be higher compared to the rest of the world (between 6.6 and 55 kg/cap).^30^ However, diets must be adjusted to meet HGD recommendations. The global average diet is ∼2950 kcal/(day*cap) with values ranging between ∼3370 kcal/(day*cap) (South and North America) and ∼2600 kcal/(day*cap) (Africa).^31^

Similarly, the MF of mobility is highly dependent on the transport modes (e.g., car, bus, or train) and their powertrain (combustion or electric engine) used to meet the service demand. Compared to the reference scenario, a transition to electric vehicles would increase the mobility MF by 26%. This is mainly because EVs require between one and four times more metals and minerals than ICEVs.^32^ Active mobility measures such as walking and cycling and reducing the use of private vehicles reduce MF. Covering short distances by walking instead of cycling (reference scenario) leads to 3% savings. Alternatively, if 23–27% (2DS and B2DS scenarios, respectively, see SI1) of the demand for private vehicles (2806 pkm/(cap*yr)) is shifted to public transport (train and bus), 10% savings can be anticipated.

### 4.4 Impacts of Collective Services in the DLS

Providing a DLS not only requires basic needs at the household level but also the provisioning of important human activities, such as recreation, social relations, and participation in society and political processes.^3^ Rao & Min^4^ argue that public space prevents overcrowding, fosters a sense of freedom, enables the pursuit of leisure activities, and allows a space to congregate for political and social activities. These needs are addressed, among others, in SDG 9.1 (develop quality, reliable, sustainable, and resilient infrastructure), SDG 10.2 (empower and promote the social, economic, and political inclusion of all), and SDG 11.7 (universal access to safe, inclusive, and accessible green and public spaces). However, so far, no attempts have been made to measure this dimension, primarily because no guidance is available in the literature for the minimum amount of space required to meet these needs.^4^ Thus, to approach this issue, we include information on additional non-residential buildings required to support human activities (see SI2). We found that the public space requirements (4.7 m^2^/cap) for a decent living standard have an MF of 454 kg/(cap*yr) (representing 8% of the total MF).

### 4.5 Cultural, Political, and Environmental Implications of the DLS

Currently, around 1.2 billion people live in multidimensional poverty (deprivations in health, education, and standard of living).^1^ Providing a DLS for these people will require a massive political effort mainly because it is not only a matter of income or even the services provided, but it is also about the required in-use stocks (direct and indirect) and the connected supply chains and environmental impacts associated. By simply scaling our results, we find that providing these people with a DLS requires an MF of about 7.2 Pg/yr and around 51.6 Pg stocks (direct and indirect).

In the second step, the DLS requirements need to be detailed for specific regions, e.g., global South and North, or even more granular, to accurately consider the stage of development, the level of urbanization, transport infrastructure, and sociocultural factors such as population size, age distribution, housing, diets, and social values that determine what a region perceives as a DLS.

The Paris agreement acknowledges the necessity of setting climate goals that safeguard development rights.^33^ However, there is a lack of articulation of what human development means under climate constraints in political debates. The DLS may serve as a baseline for defining such rights because they link human development with energy and material use and the associated climate impact and mitigation potential.^6^

### 4.6 Limitations

The major constraint of this study is the ambiguity of the service levels needed for each DLS dimension and the regional and future efficiency/technology options. Hence, the values proposed here and in other literature should be seen as first indications of DLS levels, which must to be adapted to regional cultural and climate contexts. In addition, ecoinvent has limited coverage of several stocks such as infrastructure, buildings, and machinery relevant to the DLS. Therefore, some dimensions are more accurately represented than others. For example, data for residential and non-residential buildings (hospitals, schools, offices, and others) are missing, meaning that the MF for the dimensions of shelter, education, healthcare, and collective services may be underestimated. This is mainly for two reasons. First, the data from the RECC model used to model the buildings only have information on the main material requirements, i.e., how much steel, concrete, and wood are needed per m^2^ of space, but do not have information regarding the construction or end of life (EoL) phase. Second, we only account for the buildings that are representative for each DLS dimension but do not include additional stocks needed to deliver the service in each DLS dimension, e.g., beds and medical instruments for healthcare, desks, boards, and educational equipment for education, and chairs, tables, computers, and sports and office equipment needed for collective services. Similarly, the infrastructure required to support communications, i.e., clouds, servers, and networks, is missing; thus, the MF of these dimensions may be underestimated. Similar constraints apply to the remaining dimensions (see SI2).

Second, ecoinvent has a wide geographical coverage for key processes such as electricity but is lacking for regional data for food products, water, natural gas, and many others. This implies that the analyses done at the regional level, even in the generalized form, i.e., the global South and the global North, will be based on proxy processes, mainly from Europe, that do not represent the geographical and technological conditions of the regions. These proxy may not be determinant for some DLS dimensions, such as clothing or mobility, mainly because textiles and transport technologies do not differ across regions, but they may be relevant for DLS dimensions, such as nutrition, and those that are represented by buildings, that is, shelter, healthcare, and education mainly because the geographical conditions of each region directly affect the energy consumed in the buildings either for heating or cooling and the impacts associated with food production.

Additionally, the methodological constraints of P-LCA, i.e., truncation errors by setting subjective system boundaries,^34^ the assumption of linearity, i.e., for any process, doubling the demand is associated with doubling the emissions,^35^ and temporal boundaries are present in this study. The latter particularly does not allow us to easily develop future scenarios for providing the DLS with more efficient technologies, as the processes in ecoinvent are modeled in the best case with modern technology. An alternative to tackle these issues is to conduct regionalized LCAs to customize regional processes^36^ and prospective LCAs^37^ to analyze technologies in early development stages.

Furthermore, the presented MF indicates the total mass extracted from the natural environment (TMR) and processed (RMI), which is only a rough indication of the environmental impacts of materials use, such as land degradation and energy demand for material processing. Therefore, it cannot replace more specific impact assessments, such as toxicity, eutrophication, and carbon footprint. These impacts need to be broken down into individual materials (which we do in Figure 2B) as well as regions (a limitation of P-LCA databases such as ecoinvent). Nevertheless, current and future developments may bridge this gap (see Maus et al.^38^ and Giljum et al.^39^ who used satellite image interpretation to identify regional mining tradeoff with deforestation, and Virág et al.^40^ who quantify the amount of stocks associated with mobility infrastructure).

### 4.7 Outlook

Future efforts should focus on disaggregating the results into regions. With our results, the extensive SI, and the LCA approach, own compositions of “DLS baskets” by region/country are possible. Moreover, effort should be made in estimating the amount of materials and stocks needed to close the gap between the DLS and the actual poverty as done for energy by Kikstra et al.^8^

## Supporting Information Available

S1. Method description and sensitivity analysis (PDF)
S2. Input data for the analysis (XLSX)
S3. Material footprint by unit process, environmental flows, and DLS dimensions (XLSX)
S4. Direct stocks results, indirect stocks by DLS and ISIC classification (XLSX)
S5. Results of the scenario analysis (XLSX)
