# Correction to “Material Requirements of Decent Living Standards”

**DOI:** 10.1021/acs.est.3c09400

**Published:** 2024-01-09

**Authors:** Johan Andrés Vélez-Henao, Stefan Pauliuk

A corrected version of section 4.1 appears below, in addition to corrected versions of Figures 4 and 5 (corrected caption of Figure 5). New Acknowledgment text is provided as well. Please note that, this addendum has its own reference numbers and that these presented here do not correspond with those presented in the original document.

**Figure 4:**
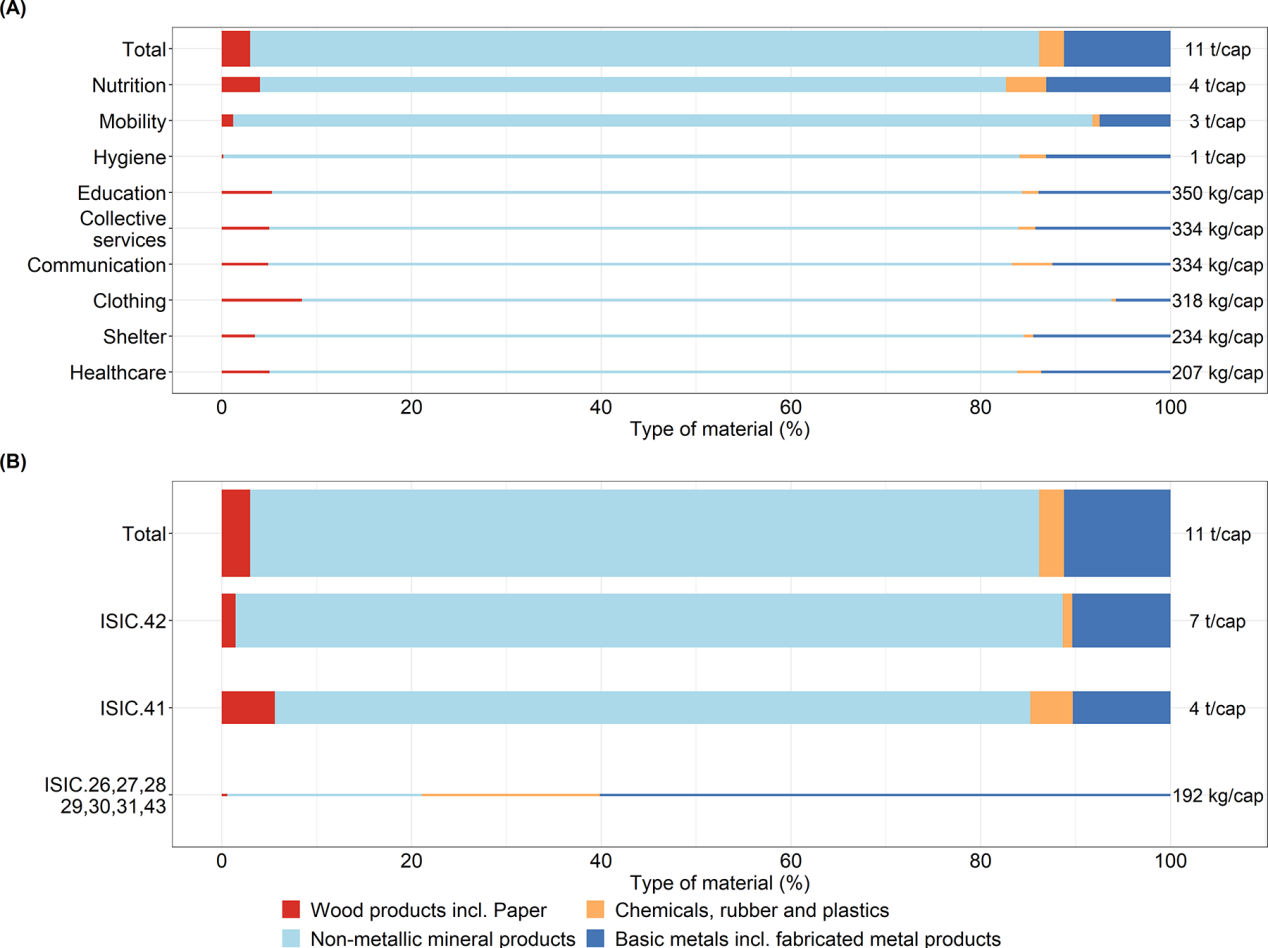
Two perspectives of the indirect stocks required to provide a decent living standard (DLS) by dimension (A) and by capital goods according to the international standard classification of economic activities (ISIC) (B). The width of each bar is proportional to the value of the total indirect stocks. ISIC 26 Manufacture of computer, electronic and optical products, ISIC 27 Manufacture of electrical equipment, ISIC 28, manufacture of machinery and equipment; ISIC 29, manufacture of motor vehicles, trailers, and semitrailers; ISIC 30, manufacture of other transport equipment; ISIC 31, manufacture of furniture; ISIC 41, construction of buildings; ISIC 42, civil engineering; and ISIC 43, specialized construction activities. The values in panel (A) are the indirect stock shown in Table 1. Data for panels (A) and (B) are provided in SI4.19–39. The results shown in the figure are calculated with eq 11 in SI1.

**Figure 5:**
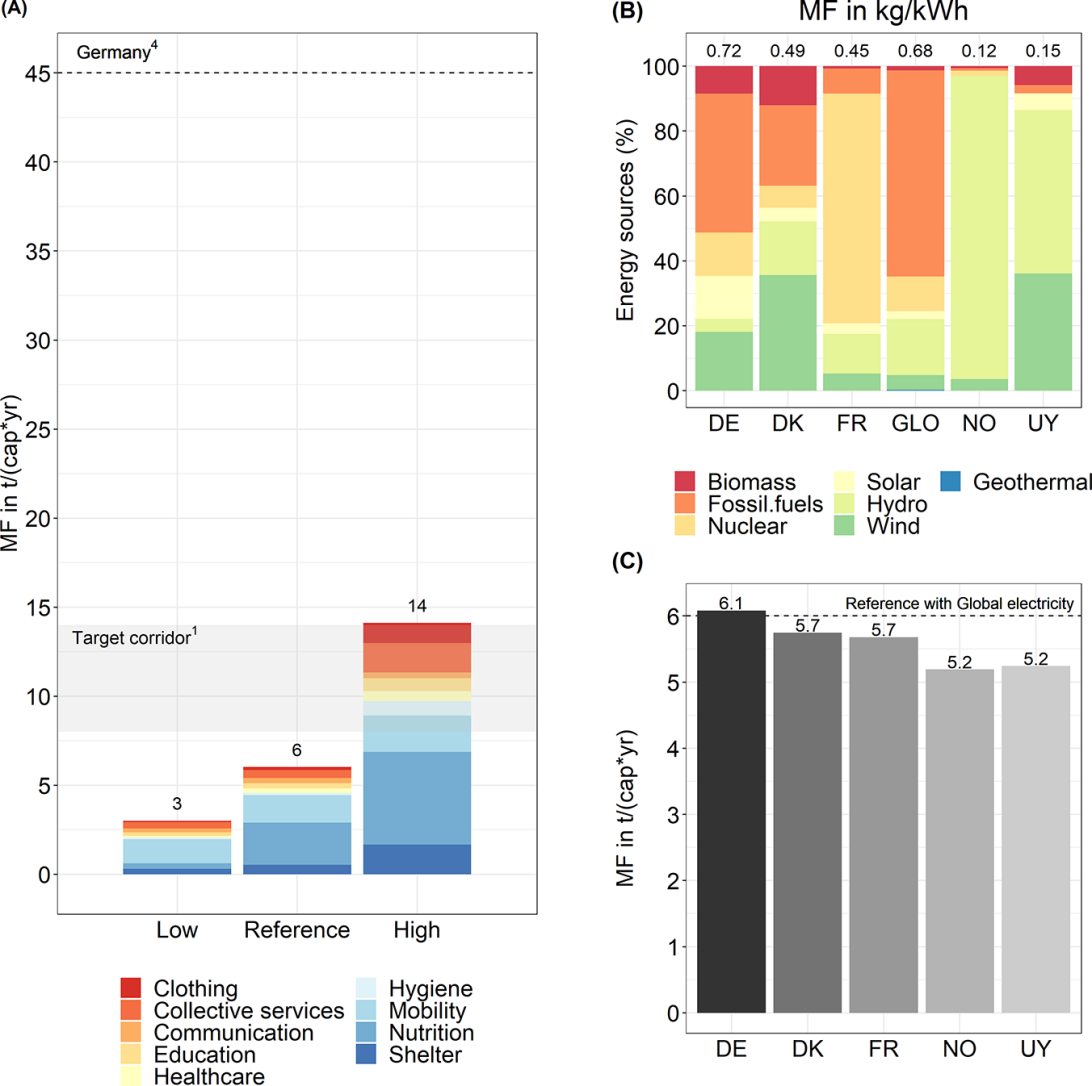
Scenario results for the total material requirement (TMR) footprint for the decent living standard (DLS), showing (A) lower and upper bound scenario results, (B) TMR by selected electricity mix in kg/kWh, and (C) TMR for DLS based on the reference scenario with different electricity mixes. For comparative purposes, the dashed line in panel A shows the total material consumption of Germany in 2008 (from ref (4) for the consumption of all final products, the TMC is the same as the MF for total material requirements^2^). The gray area in panel A represents a potential target corridor for sustainable material consumption proposed by Bringezu.^1^ The dashed line in panel C represents the TMR of the reference scenario (6 t/(cap*yr)). Data for the plots are provided in SI2.

## 4.1 DLS MF and Material Decoupling Targets

The MF (3–14 t/(cap*yr)) to provide a DLS is in line with the suggestions for a sustainable consumption of materials by 2050 found in the literature. Considering all primary resource extraction, Bringezu^1^ suggests that a potential target for achieving sustainable consumption of natural resources is given by a total material consumption (TMC) in the range of 8–14 t/(cap*yr) (for consumption of all final products, TMC is the same as the MF for total material requirements^2^). Additionally, Lettenmeier et al.^3^ suggest a TMR-based material cap of 8 t/(cap*yr) in Finland to ensure sustainable consumption of materials in the household sector.

In SI3, we report the RMI-MF that is a relevant indicator for comparison to RMC levels and corridors. Compared with current material consumption patterns, the DLS levels are much lower. For example, the RMC values associated with the United States, Germany, and China are around 30, 19, and 22 t/(cap*yr), respectively (see Bringezu^4^). The International Resource Panel (IRP) suggests that a cap on raw material consumption (RMC, excludes unused extraction) of 6–8 t/(cap*yr) is required to decouple economic growth from the use of natural resources.^5^

Furthermore, Wiedmann et al.^6^ provided information for the RMC of 186 nations for the year 2008. These results cannot be compared with the values presented in Figure 5A for TMR but with the supplementary results in SI5: around 2 t/(cap*yr) (low), 5 t/(cap*yr) (reference), and 11 t/(cap*yr) (high), respectively. For consumption of all final products, the RMC is the same as the MF for raw material inputs.^2^
