# Supplementary material for: Material Requirements of Decent Living Standards
Source: Environ Sci Technol. 2023 Sep 11;57(38):14206–17. doi: 10.1021/acs.est.3c03957 (PMC10537420; doi:10.1021/acs.est.3c03957)
Supplement: Supplementary file 1 — es3c03957_si_001.pdf [file es3c03957_si_001.pdf]

Supporting data for

Model framework, data calculation, and sensitivity analysis

Johan Andrés Vélez-Henao[†], Stefan Pauliuk[†]*

[†]Faculty of Environment and Natural Resources, University of Freiburg, Germany, 8

Tennenbacher Straße 4, 79106 Freiburg, Germany.

pages: 42, tables: 16, figures: 2

Figure S1.1 A. Global waste composition in percentage for each kg of waste, B. Global waste disposal in percentage for each kg of waste. Taken from the Word bank What a Waste 2.0.

Figure S1.2 Result sensitivity analysis, with parameter perturbations applied individually, and then together sector-by-sector (named all applied in each case).

Table S1.1 Daily food requirements (kcal/cap*day) for six age bands.

Table S1.2 Food consumption by food categories based on global dietary guidelines (HGD).

Table S1.3 ecoinvent proxy process and their energy content (kcal/gr) for each food category.

Table S1.4 Distribution of disposal waste used.

Table S1.5 Standard array of clothes assumed to be worn, along with weights, washing frequencies, and representative material.

Table S1.6 Mode-shares of pkm between transport modes.

Table S1.7 Distribution of buildings in Europe.

Table S1.8 Daily food requirements (kcal/cap*day) for a sedentary and active lifestyle. Six age bands.

Table S1.9 Daily food requirements (kcal/day*cap) and (gr/day*cap) for different diets for a moderately active lifestyle.

Table S1.10 Energy content ranges by food category.

Table S1.11 Distribution of alternative waste management scenarios.

Table S1.12 The standard array of clothes is assumed to be worn, along with weights, washing frequencies, and representative material.

Table S1.13 Electricity use in the washing and drying stages, per kg, as a function of an equipment efficiency rating.

Table S1.14 Transport mode shares for the 2DS and B2DS scenarios.

Table S1.15 The type of technologies used in each transport mode was included in the sensitivity analysis.

Table S1.16 List of parameters that are tested in the sensitivity analysis, with their reference values, low values (where applicable), and high values.

Table of Content

1. Method description......1
   1.1. Process-based life cycle assessment (P-LCA)......1
   1.2. Indirect stocks calculations......2
      1.2.1. Indirect stocks in their native units (building area, capacity)......2
      1.2.2. Indirect stocks in material units (kg)......3
   1.3. Direct stock calculation......3
      1.3.1. Direct stocks in their native units (building area, capacity)......3
      1.3.2. Direct stocks in material units (kg)......3
2. Data sources......4
   2.1. Shelter......4
      2.1.1. Household floor space......4
      2.1.2. Household buildings and thermal requirements......4
      2.1.3. Household illumination......4
   2.2. Nutrition......5
      2.2.1. Food production. 5
      2.2.2. Cooking and cold storage. 7

2.3. Hygiene 8
2.3.1. Water supply 8
2.3.2. Waste management....8
2.4. Clothing....10
2.5. Education....11
2.6. Healthcare....11
2.7. Communication 12
2.7.1. Phones 12
2.7.2. Computers 12
2.7.3. Networks and Infrastructure....13
2.8. Mobility....13
2.8.1. Total mobility requirements....13
2.8.2. Assumptions on the modal split 14
2.9. Collective services....14
3. Sensitivity analysis.....15
3.1. Shelter....15
3.2. Nutrition 16
3.3. Hygiene. 18
3.4. Clothing.....19
3.5. Education....20
3.6. Healthcare....20
3.7. Communication 21
3.8. Mobility....21
3.9. Collective services....22
4. References 27

1. Method Description

The procedure to estimate the DLS material footprint is based on four steps. First, we compile a list of services required by each DLS dimension and link them to their respective provisioning systems. Two types of provisioning system are considered: The flow-as-a-service type, such as food consumption for the service of nutrition, and the stock-operation type, where an in-use stock, such as a building, vehicle, IT device, or household appliance, is operated to provide a service to the end users. Taking nutrition as an example, the list contains flows of products such as red meat, chicken, milk, tomatoes, sugar, oil, lentils, and potatoes, energy (electricity and natural gas), and in-use stocks such as refrigerator and microwave (see supplementary information S2 for complete information of the input data used in the study).

In the second step, the reference flows for the different provisioning systems are calculated from the stock-flow-service nexus (i.e., the combination of stocks/flows needed to provide a specific service) [1]. In doing so, we rely on Rao & Min [2], Millward-Hopkins et al. [3], and the documentation therein to compile a bundle of services and stocks needed to provide a DLS in different dimensions e.g., nutrition, shelter, transport. The detail for the data sources and the list of provisioning services and reference flows by DLS dimension are provided in supplementary information S1-2, respectively.

In the third and fourth steps, we link the list provisioning systems (services as flows and in-use stocks) with the ecoinvent database to estimate the indirect stocks and material footprints.

Supplementary information S6-7 presents the indirect stocks, and Supplementary information S3-4 presents the DLS material footprints. See Figure 1 in the paper for a description of the method.

1.1. Process-based life cycle assessment (P-LCA)

To estimate the material footprint for providing a DLS we use the approach of Heijungs and Suh [4], this is rather convenient as the ecoinvent database uses the same computational structure. Thus, Heijungs and Suh first define a "technology matrix" A of flows within the economy that contains an inventory of all recorded flows in the system. Inflows to processes get a negative sign and outflows a positive sign. Additionally, they define the so-called "intervention matrix" B that records resource uptake (input -> negative sign) and emissions (output -> positive sign).

Consequently, The LCA is then formulated as follows:

$$\boldsymbol{A} \cdot \boldsymbol{s} = \boldsymbol{y} \quad (1)$$

Where A represents the technology matrix, s is a scaling vector and y represents the final demand. Thus, the scaling vector s can be determined by matrix inversion:

$$s = A^{-1} \cdot y \quad (2)$$

Similarly, the flows from/to the environment g via:

$$g = B \cdot s \quad (3)$$

Replacing s in (3) the environmental flows, for example, gangue in ground, iron in ground, can be obtained as follows:

$$g = B \cdot A^{-1} \cdot y \quad (4)$$

Moreover, to translate the environmental flows g for a given vector of demand y into material footprints, g needs to be multiplied for a matrix of characterisation factors C as follows:

$$MF = C \cdot g \quad (5)$$

Replacing (4) in (5) allows us to estimate the material footprint as follows:

$$MF = C \cdot B \cdot A^{-1} \cdot y \quad (6)$$

Where the MF is the material footprint for a given vector of demand. Thus, to estimate the material footprint for providing a decent standard living we use following set of equation for the material footprint in different layers:

$$MF\ by\ provisiong\ system = C \cdot B \cdot A^{-1} \cdot \hat{y} \quad (7)$$

$$MF\ by\ environemntal\ flow = C \cdot (\widehat{g}\,) \quad (8)$$

Here, the hat represents the diagonal of the vector of y or g, respectively. This gives us the MF in two layers, first, (7) the MF for each provisioning system required to satisfy each DLS dimension and (8) the MF by each environmental flow. Moreover, it is worth mentioning that the values in

vector y are given in services units, for example, kWh for electricity, pkm for transport, and m^2 for buildings.

1.2. Indirect stocks calculations

1.2.1. Indirect stocks in their native units (building area, capacity)

The indirect stocks can be computed from (2) by using the following procedure: First, the resulting vector s (total requirements of goods and services to provide the DLS) is filtered to extract the indirect stocks (stocks different from the in-use stocks for providing the DLS e.g., refrigerator, washing machine, vehicles). In ecoinvent, each unit process is classified by industries according to the international standard classification for industries (ISIC[i]), in this case, the ISIC codes 26 to 31 and 40 to 43 that account for capital goods in the manufacturing and construction sector. Thus, we create a new vector $\hat{s}$ to indicate the vector s with all the unit processes with ISIC codes different to capital goods (26 to 31 and 40 to 43) set to zero. Here we also set to Zero all unit processes that account for markets as these do not deliver any particular services.

As the values in $\hat{s}$ are given in flow units i.e., pkm/yr, m²/yr kg/yr. The second step is to convert them into stock units i.e., pkm, m², kg. Thus, we multiply each unit process in $\hat{s}_i$ by their respective lifetime (α_i). Where i indicates each unit process in $\hat{s}$. This information is provided by ecoinvent for most of the unit process. In the case where no lifetime is provided, we use a lifetime

[i] https://ilostat.ilo.org/resources/concepts-and-definitions/classification-economic-activities/

for a similar process as a proxy. In the case that no proxy was easy to find, we use an average lifetime of 25 yrs for machinery, 4.25 yrs for the manufacture of computers, and 15 yrs for the manufacture of motor vehicles (ISIC classification 28, 26, and 29 respectively). The vector of unit process $\hat{s}$ with their respective lifetime incl. Assumptions are provided in supplementary information S7.1. Thus, the new vector containing the indirect stock ($\tilde{y}$) can be estimated by the following equation.

$$Indirect\ stocks\ in\ native\ units = \tilde{y} = \hat{s} \cdot \alpha \quad (9)$$

Where $\tilde{y}$ accounts for a vector of indirect stocks in their native units i.e., units for buildings and km for road constructions.

1.2.2. Indirect stocks in material units (kg)

At this point, the stocks are given in units of industrial stocks, for example, unit of building, km of road, and therefore need to be converted into materials i.e., kg of aluminum and cement. Thus, we multiply the $\tilde{y}$ by the A matrix to translate into material units i.e., kg of copper, kg of plastic.

$$\boldsymbol{Indirect\ stocks\ in\ material\ units = \bar{y} = A \cdot \tilde{y}} \quad (11)$$

Once the indirect stocks have been transformed from native units, to material units it is necessary to filter by the target materials chosen in this study. We use the ISIC classification for wood and paper products, plastics, chemicals, basic metals, non-metallic metals, and fabricated metal

products (ISIC classification 16, 17, 20, 21, 22, 23, 24, 25). We also filter some products that are under the desired classification, but that account for services. For example, injection moulding, extrusion, and rolling services. To do so, we set to zero all products in the vector $\bar{y}$ different from the desired ISIC classification. Supplementary information S6-7 presents the indirect stocks in material units by DLS dimensions and ISIC product types, respectively).

1.3. Direct stock calculation

1.3.1. Direct stocks in their native units (building area, capacity)

The direct stocks are estimated similarly to the indirect stocks with the difference that this time we filter from s in (2) the direct stocks (in-use stocks of the provisioning system for the DLS). Similarly to indirect stocks, the direct stocks contained as s are given in units of flows (units/yr). Thus, each direct stock in $\hat{s}_i$ is multiplied by their respective lifetime (α_i). Where i indicates each unit process in $\hat{s}$.

$$Direct\ stocks\ in\ native\ units = \bar{\bar{y}} = \hat{s} \cdot \alpha \quad (12)$$

Where $\bar{\bar{y}}$ accounts for a vector of direct stocks in their native units i.e., pkm for vehicles or units for appliances.

1.3.2. Direct stocks in material units (kg)

The direct stocks are given in units of industrial stocks, e.g., units of phone, laptop, and therefore need to be converted into materials i.e., kg of aluminium, cement. Thus, we multiply the vector $\bar{\bar{y}}$ by the A matrix to translate into material units i.e., kg of copper, kg of plastic.

$$\boldsymbol{Direct\ stocks\ in\ material\ units} = \check{\boldsymbol{y}} = \boldsymbol{A} \cdot \bar{\bar{\boldsymbol{y}}} \quad (13)$$

We use the same ISIC classification for wood and paper products, plastics, chemicals, basic metals, non-metallic metals, and fabricated metal products (ISIC classification 16,17,20,21,22,23,24,25) used for the indirect stocks and filter the services that are under the desired classification. To do so, we set to zero all products in the vector $\check{y}$ different from the desired ISIC classification. Supplementary information S4 presents the direct stocks in material units by ISIC product types).

2. Data Sources

2.1. Shelter

2.1.1. Household Floor Space

The departure point for the analysis is the definition of the minimum requirements of space (m^2) per person and the number of persons per household. We follow the assumption made by Millward-Hopkins et al. [3], thus we assume a minimum space requirement of 10 m^2/capita plus 20 m^2/household for kitchen and bathroom facilities. In addition, we assume a household size of four persons. That gives us a total floor space requirement of 15 m^2/capita. Similar assumptions for energy requirements analysis are made by Rao & Min [2], whereas Grubler et al. [5] assume a minimum requirement of 30 m^2/capita.

To match the assumption made by Millward-Hopkins et al. [3] and for simplicity, we assume that urban and rural areas have the same floor space requirements.

2.1.2. Household Buildings and thermal requirements

The building construction archetypes are taken from the RECC database [6], the RECC model provides information for 52 different types of residential buildings, and 96 non-residential buildings in 20 regions. Data for the building archetypes contain information on the main construction materials (e.g., concrete, steel, wood, paper & cardboard) in kg/m^2 as well as energy consumption for cooling, heating, illumination, and domestic hot water (DHW) in MJ/m^2 based

on the different climate zones of the regions. We take from the RECC database the information for the construction materials and the energy consumption for cooling and heating, while for illumination and DHW we perform on our estimations. Additionally, it is assumed that buildings have a lifetime of 80 yrs.

A key innovation of RECC is the upscaling of representative descriptions ('archetypes') with different degrees of material and energy efficiency. The product archetypes were simulated with engineering tools that model building energy balance and vehicle driving cycles [7].

We take information from the RECC database for a standard single-household family in France as a representation of a temperature zone. we also assume that the heating is provided by gas while cooling is provided with electricity.

2.1.3. Household illumination

We build on the Millward-Hopkins et al. [3] approach and assumptions. To estimate the illumination requirements, it is necessary to acknowledge four key parameters: (i) how much space is illuminated, (ii) for how long each day is illuminated, (iii) how brightly, and (iv) how efficient the process of converting energy into illumination is. Millward-Hopkins et al. [3] Estimations are based on the following equation.

$$\text{Energy use} = t_{ill} \cdot \text{A} \cdot \text{E/efficacy},$$

where t_{ill} is the period of illumination (seconds/yr) A is the average floor area illuminated during this time (m^2), E is the illuminance (lm/m^2) and efficacy gives the efficiency in lm/W. The energy use here is thus in J/yr.

For A we take a value of 15 m^2/capita and assume that 33% of the space is illuminated. We also take from Millward-Hopkins et al. [3] the assumption that illumination is needed 6 hours/day. Thus t_{ill} = 7.9 million seconds (6 x 60^2 x 365) and illumination minimum level required is 125 lm/m^2. Finally, for the efficacy, we take a value of 150 lm/W.

Thus, the energy requirement is ≈ 33 MJ/cap/yr (≈ 7,9 x 15 x 33% x 125/150). We increase this to 36 MJ/cap/yr, to match the value used by Millward-Hopkins et al. [3]. Finally, to consider the capital goods needed to provide the services of illumination, we assume for simplicity and in the absence of more accurate data in ecoinvent that illumination is provided by a compact fluorescent lamp with a lifetime of 1 yr and that one lamp provides the required illumination. We do not assume any energy improvements in illumination given the minor contribution of lighting to total energy use.

This translates into a total raw material input (RMI all materials) of 449.07 kg/(capita*yr) to provide the shelter service. Whereas, for the total material requirements (TMR all), the footprint accounts for 530.03 kg/ yr*capita.

2.2. Nutrition

2.2.1. Food production.

To estimate the material requirements of food production at all stages of the supply chain 'up to the consumption phase" three parameter needs to be defined:

a. An estimate of the global average food requirement (kcal/person/day).

b. The average dietary composition.

c. The material requirements (material footprint) for producing different types of food (kg of a given material footprint/ kg of a given food product).

We follow the same stepwise procedure applied by Millward-Hopkins et al. [3]. First, we obtain the daily calorie requirements per person from the *Dietary Guidelines for Americans: 2020-2025*[ii], for 30 different age bands (from 2 to 76+ yrs of age). The daily calorie requirements are given by sex (male, female) and activity levels:

- Sedentary: A lifestyle that includes only the physical activity of independent living.
- Moderately active: A lifestyle that includes physical activity equivalent to walking about 1.5 to 3 miles per day at 3 to 4 miles per hour, in addition to the activities of independent living.

[ii] See https://www.dietaryguidelines.gov/resources/2020-2025-dietary-guidelines-online-materials (accessed 08.08.2022)

- Active: A lifestyle that includes physical activity equivalent to walking more than 3 miles per day at 3 to 4 miles per hour, in addition to the activities of independent living.

We focus on the central moderately active data as a reference point (see Table S1.1). All data was aggregated in six age groups; then we average across sex according to the proportion of male/female and the proportion of the global population by the respective age band and from the *UN World Population Prospects 2022*[iii]. Here we take the projection for 2023 in a scenario of low fertility as a reference[iv], as the growth rate of the global population is slowing.

Table S1.1: Daily food requirements (kcal/cap*day) for six age bands

Age band	moderately_active	% Total population in the age band
0-4	1,234.2	7.9
5-9	1,561.2	8.5
10-14	1,849.1	8.4
15-19	2,411.7	7.9
20-49	2,348.7	42.1
50+	2,058.3	25.2
Weight average	**2,083.5**	

Source: Dietary Guidelines for Americans: 2020-2025 and World Population Prospects 2022 UN

Estimating the dietary composition is particularly challenging due to the diet variations across the nations. As Millward-Hopkins et al. [3] stated, culture plays an important role in the composition of the persons, with high meat consumption in countries such as the United States and low meat consumption in countries such as India where religious beliefs partially or completely forbid the

[iii] See https://population.un.org/wpp/Download/Standard/Population/ (accessed 08.08.2022)

[iv] See https://www.un.org/development/desa/pd/content/World-Population-Prospects-2022 (accessed 18.11.2022)

consumption of meat. Despite that, we chose a standard diet, specifically, the dietary based on the global dietary guidelines (HGD) provided by Springmann et al. [8]. The daily intakes (g/day) of the different food categories are presented in Table S1.2. To estimate the amount of food (e.g., kg read meat, fruit & vegetables) needed to satisfy the daily energy food requirements (kcal/day) of each lifestyle, we use the *FAO's Food Balance Sheets*[v].

Table S1.2. Food consumption by food categories based on the global dietary guidelines (HGD)

	**HGD**
Red meat	33
Poultry	50
Dairy	260
Eggs	26
Fruit & Veg	492
Sugar	45
Oils	43
Pulses	16
Staples	391
total intake (gr/day)	**1,356**
Total energy intake (kcal/day)	**2,243**

Source: Springmann et al. [8]

[v] See https://www.fao.org/3/X9892E/X9892e05.htm#P8217_125315 (accessed 08.08.2022)

To select an appropriate representative product for the different food categories, we set two criteria. First, the product must be commercialized and consumed worldwide, and second, there is a correspondent global market in the ecoinvent 3.8 for the product. With these two criteria, we select the following basket of products: Boneless beef, chicken, cow milk, tomatoes, sugarcane, soybean oil, lentils, and potatoes as a fair representation of red meat, poultry, dairy, fruits & vegetables, sugar, oils, pulses, and staples, respectively, for the references model (see Table S1.3).

Table S1.3. ecoinvent proxy process and their energy content (kcal/gr) for each food category.

product category	Ecoinvent process	kcal/gr
Red meat	Market for red meat, live weight	1.5
Poultry	Market for chicken for slaughtering, live weight	1.22
Dairy	Market for cow milk	0.61
Fruit & Veg	Market for tomato, fresh grade	0.17
Sugar	Market for sugar, from sugarcane	0.3
Oils	Market for vegetable oil, refined	8.84
Pulses	Market for lentil	3.46
Staples	Market for potato	0.67

Source: Ecoinvent [9], *FAO's food balance sheets iii,* Springmann et al. [8]

Transport information is already included in the data, as we use unit process in ecoinvent with global coverage (global geography). Moreover, it is worth mentioning that the values of the eggs in each diet were not considered, since there is no data in ecoinvent to model it.

This translates into a total raw material input (RM all materials) of 1,494 kg/(capita*yr), and a total material requirement (TMR all) value of 2,279 kg/(capita*yr).

2.2.2. Cooking and cold storage.

Higher and lower GDP countries use energy differently, with the former typically relying on energy-efficient cooking appliances, while the latter typically relying on inefficient cooking appliances. We follow Millward-Hopkins et al. [3] procedure to estimate the energy consumption for cooking and refrigerating. The departure point is the range of 5-7 MJ/kg suggested by the use of FAO [10] for the energy in cooking and a nominal value of 1,500 kcal/kg for the cooked food used by Millward-Hopkins et al. [3]. Assuming an energy value of 6 MJ/kg, the energy used in cooking translates into 4 KJ/kcal (6 MJ/kg /1,500 kcal/kg) (this already includes the energy requirements for hot water in cooking). Moreover, we follow the assumptions of Millward-Hopkins et al. [3] assumptions and estimate that 50% of the calories of food calories are cooked. We assume that cooking is conducted with gas. Similarly, for cold storage, we follow Millward-Hopkins et al. [3] and assume that refrigeration requires 120 KWh/yr.

Finally, to account for the capital goods needed to provide the services of cooking and food storage, we include information for one refrigerator and one stove, with a lifetime of 10 and 15 yrs, respectively, as representation for the cooking appliances.

This translates into a total raw material input (RMI all materials) of 77.55 kg/(capita*yr) and a total material requirement (TMR all) of 88.59 kg/(capita*yr).

2.3. Hygiene

2.3.1. Water supply

We use the data from Gleick [11] to estimate the water requirements. The authors suggest 50 L/day*capita distributed in four types of home water use: 10 L for cooking, 5 L for drinking, 20 L for sanitation, and 15 L for bathing. This translates into an annual water requirement of 18,250 L/yr*capita. We use the unit process of the *market group for tap water (GLO)* and the unit process of the *market for wastewater, from residence (ROW)* to account for the provision and treatment of the water. We assume that the water provided is equal to the water to be treated. Data in ecoinvent for tap water is given in kg, while for the treatment they are provided in m^3. Here, we assume that 1 kg = 1 L and 1 m^3 = 1,000 L.

For water heating, we assume that only 25% (5 L/day*capita) of the water for sanitation purposes is heated; the remaining 15 liters is for flushing toilets. In addition, we assume that all the water required for bathing is heated.

To estimate the energy required to heat 20 L/day*capita (7,300 L/yr*capita) we use the approach used in Millward-Hopkins et al. [3]. For doing so, we need to define the initial and target temperature of the water (T_{in} and T_{out}) and heat transfer efficiency by applying the following equation.

$$\textit{Energy use} = C_p V \rho (T_{out} - T_{in}) / \textit{Eff},$$

where V is the volume of water and ρ the density (0.997 kg/L), C_p is the specific heat capacity of the water (4,184 J/kg.K) and *Eff* is the heat transfer efficiency of the boiler. Taking *Eff* = 95%, an intensity of 4,390 J/L/K is obtained. This value is multiplied by the total hot water demand (7.300 L/yr*capita) and the temperature parameters. Taking T_{out} = 50°C and an annual global average temperature of 10.7 (France as climatic temperature) as T_{in} we obtain an energy requirement of ≈ 1.3 GJ/yr*capita. Additionally, we assume that natural gas is used for water heating services. Thus, taking a calorific content of 39 MJ/ M^3 for the natural gas, we obtain a total gas requirement of 32.3M^3/yr*capita.

This results in a total raw material input (RMI all materials) of 50.7 kg/(capita*yr) and a total material requirement (TMR all) of 58.7 kg/(capita*yr).

2.3.2. Waste Management

The starting point to estimate the material footprint for residential waste management is the report of the *Word bank What a Waste 2.0*[vi]. The report provides information about waste production (kg/day*capita) by regions (e.g., North America, Europe, and, Central Asia) and income levels (e.g., low, high) for the6. According to the report, a total of 0.74 kg/day*capita of waste is generated worldwide, while national waste rates fluctuate widely from 0.11 to 4.54 kg/day*capita. Waste generation volumes are generally correlated with income levels and urbanisation rates. The

[vi] https://datatopics.worldbank.org/what-a-waste/ (accessed 10.08.2022)

global composition and waste disposition are presented in Figure S1.4. We take the global average value of 0.74 kg/day*capita as a representation of the world.

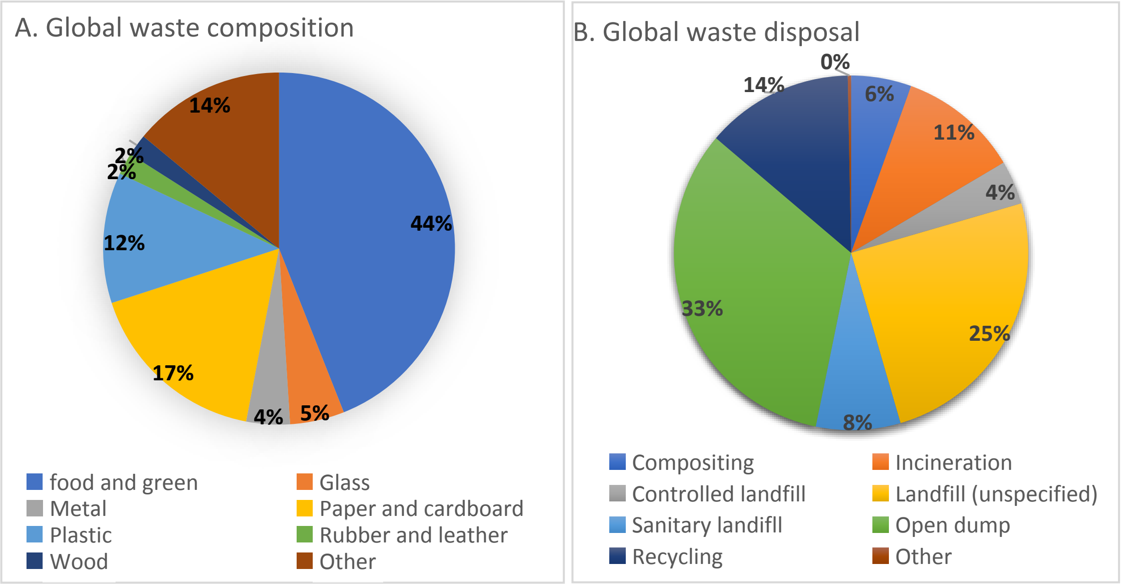

Figure S1.1. A. Global waste composition in percentage for each kg of waste, B. Global waste disposal in percentage for each kg of waste. Taken from the Word bank What a Waste 2.0.

The second step is to link the information with the appropriate unit process in ecoinvent. Ecoinvent has detailed information for different waste treatments (e.g., landfill, incineration, open dump) and types of waste (e.g., plastic, glass, paper). Thus, for simplicity we assume that: (i) the waste composition is similar to that provided for the unit process of *treatment of municipal solid waste*: paper (paper, mixed cardboard) (29%), plastic (plastics, laminated materials, laminated packaging) (17%), metals (inert metals, volatile metals, laminated materials) (6.65%), food and green (compostable material, natural products) (31%), glass (3%) and others (combined goods e.g., diapers, batteries, electronic goods, minerals) (13,35%). (ii) for waste disposition we assume

that recycling, controlled landfill, and others can be represented by the process of *treatment of municipal solid waste, sanitary landfill.* Whereas, the remaining waste treatment alternatives are represented by their respective processes in ecoinvent. In particular, ecoinvent has information on products that are partly based on recycled products, but it does not have information on the recycling process. Therefore, we assume that the recycling shares in Figure 1.B are assigned to the process of sanitary landfill process. Table S3 summarises the data used to estimate the MF for waste management.

We acknowledge that the values used and provided by ecoinvent for the waste composition of solid waste differ from those reported by the word bank (see Figure S1.1). Particularly, for the food & green which is 13% lower than the values of the Word bank. However, it offers us the opportunity to model the waste disposal in a quite decent manner (see Table S1.4).

Table S1.4. Waste disposal distribution used. Based on data from *Word bank What a Waste 2.0*

Disposal treatment	%	Value (kg/day*capita)
Open dump	33%	0.24
Incineration	11%	0.08
Landfill unspecified	25%	0.19
Composting	5%	0.04
Sanitary landfill	26%	0.19
Total	100%	0.74

This translates into a total raw material input (RMI all materials) of 47.3 kg/(capita*yr) for waste treatment. For the total material requirements (TMR all), the value is 51.4 kg/(capita*yr).

2.4. Clothing

Clothing requirements correlate with climate variation (e.g., warm countries may require fewer clothes than in cold countries, but may be washed more frequently than in cold countries due to hygiene reasons). However, we assume a standard wardrobe at global level to (i) match the assumptions made by Millward-Hopkins et al. [3] and (ii) simplicity.

Our departure point is the basic clothing list provided by Millward-Hopkins et al. [3] (see Table S1.5). The data compile information on the weight, average days worn per wash, and main material composition. The amount of clothes that are washed in a yr is the product of the weight of the clothes by the number of times that the respective clothes need to be washed in the course of a yr. E.g., for the top is 0.25 * (365/3) = 30.42 kg/yr.

Table S1.5. Standard array of clothes assumed to be worn, along with weights, washing frequencies, and representative material.

	Weight (kg)	Days/ wash	Wash(kg)/yr	material
Tops	0.25	3.00	30.42	cotton
Bottoms	0.50	15.00	12.17	cotton
Jackets	0.50	25.00	7.3	wool
Jumpers	0.50	15.00	12.17	wool
Underwear	0.05	1.50	12.17	cotton
Shoes	0.60	60.00	3.65	rubber

Total	2.4	119.5	77.89

Source. Adapted from Millward-Hopkins et al. [3]

The second step is to determine the energy consumption per wash and dry. We use data provided by Steinberger et al. [12]. The authors provide information on the energy consumption for washing for different water temperatures and efficiency rates. We used data for 60 ˚C and the efficiency rate C as a fair representation of the globe. The efficiency rate C was chosen mainly based on the data provided by Gooijer & Stamminger [13] for Europe 0.16 kWh/kg (average estimate for all the countries presented). We also assume that the energy consumed for washing in low-middle-income countries, using less efficient washing machines, is double of the consumed in Europe, 0.33 kWh/kg. We use this value as a representation of the globe, mainly 60% of the countries in the world are low-middle economies[vii].

Finally, to estimate the MF we assume that 50% of the cotton clothing (tops, bottoms, underwear) are made of cotton woven and the remaining are made of cotton knit textile. We also include information on the capital goods (wash and dried machine). Both machines have a lifetime of 10 yrs. We use the unit process for the market for washing machines for washing machines and drying machines. i.e., we assume that the production of a dryer machine is similar to the production of a washing machine. Information about water consumption is not considered since it was already included in the hygiene dimension.

[vii] https://datatopics.worldbank.org/world-development-indicators/the-world-by-income-and-region.html (accessed 15.08.2022)

This translates into a total raw material input (RMI all materials) of 123.7 kg/(capita*yr), and a total material requirement (TMR all) of 160.3 kg/ yr*capita.

2.5. Education

The estimation of the material footprint for education is based on four factors: (i) the floor-space requirement per student, (ii) the proportion of the population in education age, (iii) the material requirement for the infrastructure, and (iv) the source used to supply the heating service.

For (i) Millward-Hopkins et al. [3] assume that a student requires as much space floor for schooling as for space living (10 m^2/pupil). Whereas, for (ii) they assume that children from 5-19 age are in school (≈25% of the global population). Finally, for (iii) we use the building construction archetypes from the RECC database [6] as we did in the shelter dimension. For this means, the archetype for non-residential buildings (education) standard for a climate region (France) was taken. Here, we include the energy consumption for DHW from the RECC, as data for water consumption for education is not available. Moreover, we include the illumination service following the same procedure as in the shelter. We assume that the required space is fully illuminated 12 hour/day, since extracurricular activities like sports and working group usually take place at school after regular hours. Finally, from Cullen et al. [14] we take the illuminance level of 430 lm/m^2 for offices as a reference value.

Following the calculations made in section 2.3, we obtain a value for t_{ill} = ≈ 16 million seconds (12 x 60^2 x 365) and a total energy requirement of ≈113 MJ/cap/yr (≈ 16 x 2.4 x 100% x 430/150). Finally, as done in shelter, we include information for ecoinvent for a compact fluorescent lamp to account for the capital goods of the illumination service. In this case, we assume that 4 lamps provide the required illumination.

Finally, for (iv), we assume that the heating is provided by gas. This translates into a total raw material input (RMI all materials) of 245.4 kg/(capita*yr) and a total material requirement (TMR all) of 293 kg/(capita*yr).

2.6. Healthcare

Similarly to education, the healthcare MF depends mainly on three factors: (i) the floor space requirement per hospital bed, (ii) the average number of hospital beds per capita, (iii) the energy intensity of the floor space, and (iv) a simple upscaling to account for energy use beyond hospitals themselves. For (i), Millward-Hopkins et al. [3] provide two values (≈180 m^2[viii] and ≈200 m^2[ix]). We use the latter as a reference. For (ii) we take a value of 8 beds/1,000 citizen which translate into 1.6 m^2/capita as done by the same authors. For (iii), we follow roughly the same assumptions as made in education. The non-residential building (health) standard for a climate temperature (France) is taken as a reference. Moreover, we also include the illumination service with the

[viii] See: https://www.healthcaredesignmagazine.com/trends/research-theory/8-considerations-benchmarking/

[ix] See: https://www.energystar.gov/ia/business/tools_resources/target_finder/help/Space_Use_Information.htm

following assumption: the space is fully illuminated for 24 hours/day with an illuminance level of 430 lm/m^2 for offices as a reference value.

Thus, following the calculations from section 2.3 this gives us a value for t_{ill} = ≈ 31.5 million seconds (24 x 60^2 x 365) and a total energy requirement of ≈180 MJ/cap/yr (≈ 16 x 1.6 x 100% x 430/150). Finally, as done in shelter, we include information for ecoinvent for a compact fluorescent lamp to account for the capital goods of the illumination services. In this case, we assume that 4 lamps provide the required illumination.

For (iv), we assume that the heating is provided by gas. This translates into a total raw material input (RMI all materials) of 205 kg/(capita*yr), and a total material requirement (TMR all) of 255 kg/ yr*capita.

2.7. Communication

2.7.1. Phones

Rao & Min [2] suggest that one phone per adult is required for a decent living. We follow the Millward-Hopkins et al. [3] procedure and assume one phone per person (over the age of 10 yrs). Taking the data for the *UN World Population Prospects 2022*[x], this translates into a value of 0.83 phones/cap for the global population. We use the unit process of the *market for consumer electronics, mobile devices, and smartphone* from ecoinvent to account for it. This data set

[x] See https://population.un.org/wpp/Download/Standard/Population/ (accessed 08.08.2022)

provides information on a Fairphone 1, a mid-range smartphone from 2014 with a 4.3 inch display and a total weight of 163.45gr with a lifetime of 2.5 yrs [9].

Different values can be found in the electricity consumption of the literature for the in-use phone. Grubler et al. [5] provide values in the range of 5-1 watts at full power and standby, respectively. Assuming a use of 16 hours, this translates into 5.8 to 29.2 kWh/yr. Ercan et al.[15] suggest values for three different uses. Light (2.6 kWh/yr), average (3.9 kWh/yr), and heavy (7.7 kWh/yr). Moreover, an LCA report for the Fairphone 2 suggests a yearly consumption of 4.9 kWh [16]. We use the higher value provided by Ercan et al.[15] as a reference, mainly because according to Grubler et al. [5] phones replace several electronic functionalities; therefore, an intensive use of phones.

2.7.2. Computers

Rao & Min [2] suggest that one laptop is required per household for a decent living. This translates into a value of 0.25 laptops/per capita. We use the unit process of the *market for computers and laptop* from ecoinvent to account for it. This data set represents a laptop with a processor speed of 600 MHz, 10 GB RAM, 128 MB memory, a 12.1 inch screen, and a total mass with an expansion base of 3.15 kg with a lifetime of 4.25 yrs [9].

Regarding electricity consumption. We take the value of (62 kWh/yr) provided by Deng et al. [17].

2.7.3. Networks and Infrastructure

We include the unit process *market for routers, internet* as a representation of the capital goods needed to provide and support telecommunications. This unit process represents a domestic router with a maximum data rate of 100 Mbit/s and a realistic data rate of 25 Mbit/s and a useful life of 6 yrs [9]. We assume one router/household or 0.25/capita.

Regarding electricity consumption, we use the data provided by Ercan et al.[15] for Wi-Fi and phone operation networks. The provided values are 49 kWh / year (heavy use), 33.3 kWh/year (average use), and 28.7 kWh/yr (light use). For laptops, we follow Millward-Hopkins et al. [3] assumptions and take a value five times higher than the electricity required for networks and Wi-Fi for a phone that is used heavily. In total, we estimate a total electricity consumption of 364.4 kWh/yr for the provision of the internet.

This translates into a total raw material input (RMI all materials) of 238.4 kg/(capita*yr) for waste treatment. Whereas, for the total material requirements (TMR all) the value is 293.5 kg/capita*yr.

2.8. Mobility

2.8.1. Total mobility requirements

Grubler et al. [5] suggest a minimum of 7,000 km/capita*yr for mobility based on the results obtained for Japan, an affluent society with an advanced transportation system. Alternatively, the two-degree scenario (2DS) suggests average annual mobility levels in 2025 ranging from <5,000

km/cap in India to >20,000 km/cap in the USA. While, the world average annual mobility in the world will increase from ~7,000 km/cap in 2014 to ~10,000 km/cap by 2060 [18].

We take these values as a reference and follow the Millward-Hopkins et al. [3] approach to estimate the annual mobility requirements per capita (pkm/yr*capita):

$$MOB = MOB_{base} * (f_{fixed} + f_{variable} \times LD_{base}/LD),$$

Where:

LD = Population density = Population Density × Total land area / Inhabited land area,

Inhabited land area = Agricultural land area + urban land area.

Where MOB is the annual mobility requirements per capita (pkm/(yr*capita)), (f_{fixed}) represents a proportion of mobility that is *independent* (travel that is independent of local population density), ($f_{variable}$) is inversely proportional to population density, and (LD) is the lived density. (LD_{base}) represents the global median lived density (LD_{base} = 189 persons/km^2). Finally, it is assumed that (fixed) and ($f_{variable}$) shares the same proportions (50%-50%).

To estimate LD we use the global density information for 2010 provided by the World Bank Database[xi]. This gives us a value of 138.6 (53 capita/ km^2*1.32E+08 km^2 / (4.7E+07 km^2 + 3.6E+06 km^2)). This gives a total mobility requirement of 8,274 km/capita*yr.

[xi] https://data.worldbank.org/?name_desc=true (accessed 19.08.2022)

2.8.2. Assumptions on the Modal Split

The next step is to specify the share of transport modes. We divided transport modes into five categories (non-motorised transport, air transport, rail transport, road transport buses, road transport-cars, and road transport-2 wheelers). For non-motorised transport (e.g., walking or biking), we use the value provided by Millward-Hopkins et al. (2020) (4 km/capita*day or 1,460 km/capita*yr). For the rest of the transport modes, we use the values of the 2060 reference technology scenario (RTS) provided by IEA [18] (see Table S1.6).

Table S1.6. pkm between transport modes

Mode	RTS shares (%)	pkm/(capita*yr)
Walking-cycling		1,460
2-wheelers	12%	841.1
PLDVs	41%	2,806.5
Buses	16%	1,082.9
Rail	6%	433.3
Air	24%	1,649.9
Total	100%	8,273.7

PLVD: passenger light-duty vehicles

Source: Millward-Hopkins et al. [3] and IEA [18]

The last step is to select the appropriate unit process to model each transport mode. Ecoinvent has a wide range of processes to this end. For example, for the air mode, ecoinvent has four options depending on the distance of the haul (very short, sort, medium and long). Similarly, there are different options for road transport modes (except for the train that only has the diesel option), depending on the fuel and the size of the vehicle. Thus, we made the following assumptions for

the reference scenario: (i) the 1,460 pkm associated with non-motorised vehicles is provided by a bike, (ii) for the 2-wheelers, the information for a motor scooter(petrol) is used, (iii) for buses and train, we use the information for a regular bus and a long-distance train operated with diesel, (iv) for the car, we take a medium-size car impulse by natural gas, and (v) for the aircraft, we use the information for a medium haul.

It is worth mentioning that in ecoinvent the unit reference for all transport modes different from the car is pkm, whereas for the car the unit reference is given in km. Therefore, for simplicity, we assume an occupancy rate of 1 person per car to account for the pkm by car assumed in each scenario.

This translates into a total raw material input (RMI all materials) of 1,346.28 kg/(capita*yr) and a total material requirement (TMR all) of 1,555.74 kg/capita*yr.

2.9. Collective services

We include information on additional public-government infrastructures required to support modern societies. The rationale behind this is that to promote and support a modern society different non-residential building such as wholesale & retail, offices, hotels & restaurants, and sports facilities are needed.

To account for the demand for non-residential buildings per capita, we start with the information provided by the BPIE [19] for Europe. The report compiles information for different types of

building across 30 Europe countries including the population of each country. In short, the report concludes that 25% of the total buildings in Europe are non-residential buildings used to support different activities (see Table S1.7). Here, we made two assumptions. First, we exclude the values for education and hospitals, as we already include them in their respective DLS dimension. Second, for the remaining building archetypes we take half of the non-residential requirements (4.69 = 9.40/2) as a proxy to the minimum requirements of collective infrastructures required to provide social wellbeing.

Table S1.7. Buildings distribution in Europe

Total Buildings (Billion m^2)	250		
Europe Population (Million)	512		
	total in %	m^2	m^2/cap
Residential buildings	75%		
Wholesale & retail	7%	1.75E+09	**3.42**
Offices	6%	1.44E+09	**2.81**
Educational	4%	1.06E+09	2,08
Hotels & Restaurants	3%	6.88E+08	**1.34**
Hospitals	2%	4.38E+08	0.85
Sport facilities	1%	2.50E+08	**0.49**
Other	3%	6.88E+08	**1.34**
Total non-residential building			**9.40**

We take from the RECC database the archetypes associated with each type of building for a climate temperature (France). Moreover, we also include the illumination service with the following assumption: First, the required space is fully illuminated for 12 hour/day with an illuminance level of 430 lm/m^2 for offices as a reference value.

This translates into a total raw material input (RMI all materials) of 364.8 kg/(capita*yr) and a total material requirement (TMR all) of 454.5 kg/capita*yr.

3. Sensitivity analysis.

In this section, we describe the parameters and values chosen for the DLS dimension sensitivity analysis.

3.1. Shelter

As the type of temperature zone and the building determine the demand for materials and energy in buildings, we perform a sensitivity analysis with the remaining 34 building archetypes of the RECC model. Moreover, we include information on India as a representation of a warmer zone as a proxy for Asia, where about 2/3 of the world's population lives. Finally, we also assume that all services demanded by the household, i.e., cooling, heating, and illumination, are provided by electricity.

Additionally, for illumination, we assume that the service uses twice as many lights that are used twice the time. Similar assumptions are used in Millward-Hopkins et al. [3]. Thus, the energy requirement is ≈ 130 MJ/cap/yr (≈ 15.8 x 15 x 33% x 250/150).

Summarising. We perform a total of 288 different scenarios. Hight values are obtained for a scenario in which a residential tower (RT) building located in a temperature area is used, the

heating service is supplied by electricity, and the household is highly illuminated. This scenario gives a total raw material input (RMI) of 1,339 Kg/(capita*yr), whereas for TRM the value is 1,663 kg/(capita*yr). On the other hand, the lowest values are obtained in the multi-familiar standard household residence with RES2,1 + RES2,2 (lighter and with more wood materials) located in a temperature climate (France) in which the gas is used in heating, and the household is moderately illuminated. Concretely, the results for RMI are 227 Kg/(capita*yr), whereas, for TMR the value is 300.6 Kg/(capita*yr).

3.2. Nutrition

Different parameters influence the MF of nutrition. (i) lifestyle, (ii) the type of diet, and (iii) type of food consumed to provide the required kcal/day. For (i), we include the information on a sedentary and active lifestyle from the *Dietary Guidelines for Americans: 2020-2025*[i] (see Table S1.8). All data were aggregated into six ages, then we averaged across sex according to the proportion of male/female and the proportion of the total population by the respective age band from the *UN World Population Prospects 2022*[ii].

Table S1.8: Daily food requirements (kcal/cap*day) for a sedentary and active lifestyle. six age bands

Age band	Sedentary	Active	% Total population in the age band
0-4	1,066.66	1,300.88	7.9
5-9	1,341.81	1,781.81	8.5
10-14	1,723.90	2,304.55	8.4

15-19	2,129.05	2,771.64	7.9
20-49	2,119.58	2,606.48	42.1
50+	1,844.74	2,342.42	25.2
Weight average	**1,868.44**	**2,354.24**	**100**

Source: Dietary Guidelines for Americans: 2020-2025 and World Population Prospects 2022 of the UN

Furthermore, we use two dietary recipes (vegetarian (VGT) and vegan (VGN)) provided by Springmann et al. [8] and created a new recipe with low consumption of meat (red and poultry). We use the recipe for the global dietary guidelines (HGD) provided by Springmann et al. [8] as a baseline and reduce meat consumption by 50%. To keep the kcal/day intake constant, we increase the proportion of food in the other categories in similar proportions (see table S1.9).

Table S1.9: Daily food requirements (kcal/day*cap) and (gr/day*cap) for different diets for a moderate active lifestyle

	LOWMEAT	VGT	VGN
Red meat	16.5	0	0
Poultry	25	0	0
Dairy	265.92	260	0
Eggs	31.93	26	0
Fruit & Veg	497.93	545	606
Sugar	50.93	45	45
Oils	48.93	43	43
Pulses	21.93	80	80
Staples	396.93	352	436
total intake (gr/day)	**1,356**	**1,351**	**1,210**
Total energy intake (kcal/day)	**2,243**	**2,243**	**2,243**

Source: Source: Springmann et al. [8]

For (iii) as the energy content (kcal/gr) of food dramatically varies across products and categories (e.g., for red meat: 0.77 kcal per gr of buffalo meat and 1.5 kcal per gr of Beef Boneless), the product chosen as a proxy for each food category may significantly influence the final results. Thus, we estimate the average, maximum and minimum energy content (kcal/gr) of each food category based on the *FAO's food balance sheets*[xii] (see table S1.10). Here, it is worth mentioning that, while we change the energy content (kcal/gr) of the different food products, we do not change the unit process from the ecoinvent used to estimate the material footprints of the different diets.

Table S1.10. Energy content ranges by food category

[xii] See https://www.fao.org/3/X9892E/X9892e05.htm#P8217_125315 (accessed 08.08.2022)

Product category	Ecoinvent process	Kcal/gr	Average (kcal/gr)	Max(kcal/gr)	Min(kcal/gr)
Red meat	Beef boneless	1.5	1.72	4.62	0.42
Poultry	Chicken	1.22	1.77	3.01	1.22
Dairy	Cow milk	0.61	1.71	4.96	0.26
Fruit & Veg	Tomatoes	0.17	0.63	3.46	0.09
Sugar	Sugarcane	0.3	3.07	3.9	0.3
Oils	Soybean oil	8.84	8.67	9.02	7.11
Pulses	Lentils	3.46	3.48	3.9	3.25
Staples	Potatoes	0.67	3.20	4.39	0.67
Staples (a)	Rice	3.34	3.20	4.39	0.67
Staples (b)	Wheat	2.8	3.20	4.39	0.67

Source: *FAO Food Balance Sheets*[xiii].

Additionally, as the products to satisfy a specific diet vary across cultures, e.g., rice is more consumed than potatoes in India than in Europe, we replace the potatoes for rice and wheat to provide a range of variation (including the ecoinvent unit process). Finally, we also include a microwave as a cooking appliance.

We acknowledge that this pragmatic approach may lack precision, since the supply chain of the different products may differ considerably (e.g., bananas and tomatoes). However, since we intend to provide a range of values in which the different types of diets may fall, more than provide an optimised basket of products to satisfy a determined diet, we consider this model approach a fair representation of the wide spectrum of possible results.

Additionally, to consider the energy sources and efficiency of the appliance needed to provide the service of nutrition, we prepare additional scenarios. We assume 25% or 75% of the kcal

[xiii] See https://www.fao.org/3/X9892E/X9892e05.htm#P8217_125315 (accessed 08.08.2022)

consumed is cooked, moreover, we assume cooking is conducted with electricity. According to Cullen et al. [14], the energy intensity of cooking and refrigeration could be reduced by 80%. Thus, we also consider an efficiency scenario in which services are 80% more efficient (i.e., 0.8 KJ/kcal of food for cooking and 24 KWh/yr for refrigeration).

In total, we conducted 3,456 scenarios for the nutrition dimension. High values are obtained for HGD active lifestyle with HGD in which rice is used to approach the staple. This scenario also assumes that a large number of kcal is cooked with electricity in standard devices (refrigeration and stove), and the kcal/gr of the selected products is the minimum of the available products from the FAO. This scenario gives a total raw material input (RMI) of 3,534.9 kg/(capita*yr), whereas for TRM the value is 5,213.1 kg/(capita*yr). On the other hand, the lowest values are obtained for a sedentary lifestyle, in which potatoes are chosen as a representation of the staple. Moreover, a low number of kcal is cooked with natural gas. This scenario also assumes that all the devices (microwave as cooking appliance) used are efficient and the kcal/gr of the selected products is the maximum of the available products from the FAO. Concretely, the result for RMI is 215.4 kg/(capita*yr), whereas, for TMR and TWF result is 315.6 kg/(capita*yr).

3.3. Hygiene.

The energy consumption for water heating depends mainly on three factors, the initial and target water temperature, the amount of water to be heated, and the efficiency assumed. For the former,

we used a yearly average temperature of India of 23.65 ° C as a representation of a warm land as the initial temperature and a value of 60 ° C of the target temperature. Below 65 ° C, a typical value according to Cullen et al. [14]. For the amount of water to be heated, we increase the value by a factor of 2 (14,600 lt/yr*capita) to match the recommendation of the ONU[xiv] (50-100 lt/yr*capita). Finally, for the latter, we assume an efficiency for the heating water of 98%.

Regarding waste management, there are two key parameters: (i) the amount of waste produced and (ii) the disposition technology. We use the former two values for waste production, the maximum (4.54 kg/capita/day) and minimum (0.11 kg/capita/day) values from Word bank What a Waste 2.0. For the latter, we include two future alternatives in which we increase 50% the amount of waste to be composted and incinerated, respectively, in each scenario. By doing so, we assume that the other disposition technologies different from incineration and composting decrease in equal proportions to maintain unity (see table S1.11)

Table S1.11. Distribution of alternative waste management scenarios. Data from *Word bank What a Waste 2.0.*

	World(reference)	comp50%	inci50%
Open dump	33%	32.4%	31.6%
Incineration	11%	10%	17%
Landfill unspecified	25%	24%	24%
Composting	5%	8%	4%
Sanitary	26%	25%	25%
Total	100%	100%	100%

[xiv] https://web.archive.org/web/20210213090811/https://www.un.org/en/sections/issues-depth/water/ (accessed 11.08.2022)

In total, we conducted 288 scenarios for the hygiene dimension. High values are obtained for a climate temperature area in which 40 litres/day*capita of water is heated at 60 ˚ C with electricity. Additionally, regarding waste management, this scenario is based on a daily waste production of 4.54 kg/day*capita (maximum value) in which 33% of all the waste is deposited in an open dump. This scenario gives us a total raw material input (RMI) of 665.6 kg/(capita*yr), whereas for TRM the value is 808.3 kg/(capita*yr). On the other hand, the lowest values are obtained for a warm area in which 20 liters/day*capita of water is heated at 50 ˚C with an efficient gas boiler. Moreover, this scenario includes a waste production of 0.11 kg/day*capita (minimum value) in which 17% is incinerated. This scenario provides values for RMI of 75.28 kg/(capita*yr) and for TMR of 86.98 kg/(capita*yr).

3.4. Clothing.

Four parameters determine the MF for clothing. (i) The number of clothes per capita, (ii) the material of the clothes, (iii) the days/wash of each clothes, (iv) the efficiency of the wash and dry machine, and (v) the use or not of a dryer. For the (i) parameter, we assume that the double of clothes is needed as a representation of a cold weather. For (ii), we assume that the material composition of the tops, bottoms and underwear is produced 100% by organic textile, e.g., cotton wove, cotton knit, silk, kenaf, jute, or synthetic materials, e.g., polypropylene and polyester textile. For (iii) we assume that clothes are washed less often (see table S1.12), for (iv) we include

different efficiency rates, specifically the efficiency rates A to D of Gooijer & Stamminger [13] and an alternative rate (effi) that include an improvement in efficiency of 91% for washing and 65% for drying suggested by Cullen et al. [14](see table S1.13. Finally, for (v) we assume that the dryer is not used.

Table S1.12. It is assumed to be worn, along with the weights, washing frequencies, and representative materials.

	Weight (kg)	Days/ wash	Wash(kg)/yr	material
Tops	0.5	6	60.83	1 to 7
Bottoms	1	30	24.33	1 to 7
Jackets	1	50	14.6	wool
Jumpers	1	30	24.33	wool
Underwear	0.1	3	24.33	1 to 7
Shoes	1.2	120	7.3	rubber
Total	4.8	239	155.7	

1.Cotton woven, 2. Cotton knit,3. Silk,4. Polypropylene,5. Polyester,6. Kenaf,7. Jute

Table S1.13. Electricity use of the washing and drying stages, per kg, as a function of an equipment efficiency rating.

	Equipment rating				
Washing and drying (kWh/kg)	Effi	A	B	C	D
Wash(60˚C)	0.02	0.24	0.29	0.32	0.34
Dryer	0.19	0.55	0.64	0.73	0.82

Source. Adapted from Gooijer & Stamminger [13]

In total, we conducted 320 scenarios for the clothing dimension. Hight values are obtained for the scenario in which 4.8 kg clothes/yr*capita is required. This scenario assumes that in total clothes need to be washed 156 days and that 66% of clothes are made of silk. In addition, it is assumed that the clothes are dried and that the efficiency type of both the washing and dried machine is D. This scenario gives a total raw material input (RMI) of 810 kg/(capita*yr), whereas for TRM the value is 1,135 kg/(capita*yr). On the other hand, the lowest values are obtained for a scenario in which 2.4 clothes/yr*capita is required. This scenario assumes that in total the clothes need to be washed for 77 days and that 66% of clothes are made of polypropylene. Moreover, it is assumed that clothes are dried naturally and that the washing machine is highly efficient (91% compared to type A). This scenario provides a value for RMI of 53.8 kg/(capita*yr) and a value for TMR of 71.5 kg/(capita*yr).

3.5. Education

There are four factors that affect the MF of education: (i) floor space per student, (ii) illumination level, (iii) the build infrastructure, and (iv) energy source used to provide the heating, cooling, water heating, and illumination. For (i) we assume that 15 m^2 is required per student, while for (ii) we use half of the illumination required per m^2 (215 lm) as not all rooms need to be fully illuminated and that a proportion of the buildings are hallways that required 54 lm/ m^2 [14]. Under this scenario, half of the lamps are required (2 lamps). Regarding the building, we include the 16 different archetypes for non-residential buildings (education) from the RECC model that account for different efficiency scenarios and the data for India to include a warm temperature as done in the shelter dimension. This gives us a total of 32 different types of buildings. Finally, for (iv), we assume that heating (both space and water) is provided by electricity.

This gives us a total of 256 different scenarios. High values are obtained for the case in which the building archetype non-standard,.RES0 2.2 for Indien is selected, in which 15 m^2 per student and 430 lm/ m^2 are selected. Additionally, the heating service is provided with electricity. This scenario has a total RMI of 571.7 kg/(capita*yr) and a TMR of 714.7 kg/(capita*yr). Similarly, low values are obtained in the case of the building archetype non-standard,.RES2.1 for France is selected, in which a space per student is 10 m^2 with an illumination requirement of 215 lm/ m^2 is used. In this scenario, heating is provided with gas. This scenario has a total RMI of 158.4 kg/(capita*yr) and a TMR of 197.7 kg/(capita*yr).

3.6. Healthcare

Five factors determine the MF of healthcare (i) the floor space per bed, (ii) the number of beds per citizen, (iii) the illumination levels, (iv) the building infrastructure and (v) the energy source used to provide the heating, cooling, water heating and illumination. For (i) we assume that 180 m^2 is required per bed, while for (ii) a value of 10 beds/citizen is taken, both values mentioned in Millward-Hopkins et al. [3]. For (iii) half of the illumination required per m^2 (215 lm) is used as some areas of a hospital do not require to be fully illumined all the time. Under this scenario, half of the lamps are required (2 lamps). Regarding the building, the 16 different archetypes for multi-family buildings that account for different efficiency scenarios, and the data for India to include a warm temperature as done in the shelter dimension. Finally, for (v), we assume that heating (both space and water) is provided by electricity.

This gives us a total of 512 different scenarios. High values are obtained for the scenario in which a non-standard building in a warm temperature is chosen. This scenario assumes a non-standard building RES0 in India with a bed requirement of 200 m^2, and 10 beds/1,000 citizens, the illumination required is 430 lm/ m^2, and that the heating is provided by electricity. This gives us a total value for RMI of 455.2 kg/(capita*yr) and a TMR of 568 kg/(capita*yr). Alternatively, low values are obtained in the case in which a non-standard (RES2.1) building in a warm temperature with material change is chosen. This scenario assumes that a bed requires 180 m^2, there are 8 beds/1,000 citizens, the illumination required is 215 lm / m2 and that the heating is

provided by gas. This gives us a total value for RMI of 75.5 kg/(capita*yr) and a TMR of 96.09 kg/(capita*yr).

3.7. Communication

The energy consumption of phones and laptop correlates with their daily use. Based on the data provided by Ercan et al.[15] we create two additional scenarios that correspond to average (3.9 kWh/yr) and light use (2.6 kWh/year). Thus, the electricity consumption associated with the provision of Wi-Fi is changing according to the intensity of phone use. Additionally, we assume that the electricity required to provide WI-FI and network infrastructure for laptops is ±25% of the value used in the reference scenario (245 kWh/yr).

In total, we conducted 9 scenarios for the communication dimension. Hight values are obtained for the scenario in which the phones and laptops are used intensively, whereas, the low values are those for which the devices are less used. Results for the former are on the order of 272 kg/(capita*yr) (RMI) and 335 kg/(capita*yr) (TMR). Whereas, the latter results are 192 kg/(capita*yr) (RMI), and 235 kg/(capita*yr) (TMR).

3.8. Mobility

The impacts associated with mobility are mainly determined by three factors (the amount of km/capita*yr required, the share of each transport mode to satisfy the desired pkm requirements, and the type of technology of each transport mode. We decide to modify the two latter parameters.

First, we use the 2 ° C scenario (2DS) and the Beyond 2 ° C scenario (B2DS) provided by the IEA [18](see table S1.14) to determine the shares of transport modes.

Table S1.14. Transport mode shares for the 2DS and B2DS scenario

	2DS (%)	B2DS (%)	2DS (km/capita*yr)	B2DS (km/capita*yr)
Walking/biking			1,460	1,460
2-wheelers	11%	10%	717.7	650.8
PLDVs	32%	30%	2,149.2	2,054.5
Buses	23%	25%	1,567.4	1,680.6
Rail	15%	21%	1,026	1,409.4
Air	20%	15%	1,353.4	1,018.4
Total	100%	100%	8,273.7	8,273.7

Source: IEA [18]

Second, for the transport modes we develop several scenarios, we assume that the shares of mobility provided by non-motorized vehicles can be provided by walking (in which no impacts are associated), biking (regular or E-bike), and a combination of 50-50 between walking and biking (regular or E-bike), similarly, for the 2-wheelers transport shares we include an E-scooter. For the passenger light-duty vehicles (PLDVs), four types of vehicles are included, two transport modes for the buses, and four for aircraft are also included. See Table S1.15.

Table S1.15. The type of technologies used in each transport mode was included in the sensitivity analysis. In bold reference scenario. Bold values are the baseline case

Non-motorized	2-wheelers	Car	Bus	Air
Walking (100%)	**petrol-scooter**	Diesel	**Diesel**	Very-short

**Bike (100%)**	E-scooter	Petrol	electricity	Short
E-Bike (100%)		**Natural gas**		**Medium**
Walking bike (50-50)		Electricity		Long
Walking-E-bike (50-50)				

In total, we conducted 960 scenarios for the mobility dimension. Hight values are obtained for the scenario RTS in which the electromobility predominates i.e., the use of e-bikes, electro-scooters, cars, and buses, in combination with flying short distances. This scenario gives a total RMI of 1,700 ton/(capita*yr), whereas for TRM the value is 2,037.5 ton/yr*capita. On the other hand, the lowest values are obtained in the 2SDS without use of electro-mobility i.e., walking, diesel for the scooter and bus, natural gas for the cars, and flying long distances. Concretely, the result for RMI is 1,176 ton/yr*capita, whereas, for TMR the result is 1,351 ton/yr*capita.

3.9. Collective services

Similarly to education, four factors are key in estimating the MF of collective services: (i) the floor space per capita, (ii) the illumination levels, (iii) the build infrastructure, and (iv) the energy source used to provide the heating,, cooling, water heating and illumination. For (i), we assume that 9.40 m^2 is required per capita, while for (ii) we use half of the illumination required per m^2 (215 lm). Under this scenario, half of the lamps are required (2 lamps). Regarding the building, we include the 16 different archetypes of buildings that account for different efficiency scenarios and the data for India to include a warm temperature, as done in the shelter. This gives us a total

of 32 different types of buildings. Finally, for (iv), we assume that heating (both space and water) is provided by electricity.

This gives us a total of 256 different scenarios. High values are obtained for the case in which a non-standard building is used in a warm temperature and 15 m^2 for space and 430 lm/ m^2 for illumination is selected. Additionally, the heating service is provided with electricity. This scenario has a total RMI of 1,313 kg/(capita*yr) and a TMR of 1,649 kg/(capita*yr) respectively. Similarly, low values are obtained in the case of non-standard (RES2.1) building in a template temperature selected. Additionally, this scenario assumes a space per student of 10 m^2 and an illumination requirement of 215 lm/ m^2. In this scenario, heating is provided with gas. This scenario has a total RMI of 280 kg/(capita*yr) and a TMR of 352 kg/(capita*yr).

A summary of the parameters used in each DLS dimension to construct the scenarios is presented in table S1.16, results of the scenarios by DLS dimension are presented in Figure S1.2

Table S1.16. List of parameters that are tested in the sensitivity analysis, with their reference values, low values (where applicable), and high values.

	Input parameters	lifestlyle and efficiency choices		Details
	Reference	Low material consumption	high material consumption	
**Shelter**				
Household size	4 person /hh	_	_	No change in household structure assummed
Floor space	15 m2	_	_	10 m2 per person and 20 m2 collective space
Building type	SFH.RES0 climate temperature (France)	MFH_standard,.RES2,1+RES2,2. Climate temperature (France)	SFH_standard,.RES0. Warm temperature (India)	Data for the efficiency and building materials from the RECC model
Energy source heating	Natural gas	_	Electricty	
Illumination	125 lm/m2	_	250 lm/m2	Data calculation from Millward-Hopkins et al., (2020)
**Nutrition**				
Diet	HGD	_		Four diets studied HGD; low meat (-50% from HGD), VGT,VGN
Living style	Moderate active	sedentary	active	Based on the UN World Population Prospects 2022
Kcal contend of food	Average	max values	min values	Values taken from Word bank What a Waste 2.0, average,max and min values
Energy source cooking	Natural gas	_	Electricty	
Efficiency applicances	4 KJ/Kcal	8 KJ/kcal	_	Millward-Hopkins et al., (2020), 80% effiency assumed
Amount food in kcal cooked	50%	25%	75%	Millward-Hopkins et al., (2020), low and high scenario an arbitrary +/- 25%
Staples type	Potatoes	_	rice	Given the socio cultural factors rice, potatoes and wheat were analysed
**Hygiene**				
Place temperature	Climate temperature (France)	_	Warm temperature (India)	
Heater efficiency	95%	98%	_	Value for efficiency from Cullen et al., (2011)
Temperature water out	50°c	_	65°c	Reference from Millward-Hopkins et al., (2020), 60°c arbitrary value, while 65 °c is a typical value Cullen et al. (2011)
Quantity water to heat	20 m3	_	40 m3	Reference from Gleick (1996), high scenario to meet values of ONU
Rate waste	0,74kg/(cap*day)	0,11kg/(cap*day)	4,54kg/(cap*day)	Values taken from Word bank What a Waste 2.0
Technology Wastetreatment	world average			From Word bank What a Waste 2.0, abitrary 50% increase in incineration or compost
Energy source heating	Natural gas	_	Electricty	

Table S1.16. (continued)

	Input parameters	lifestlyle and efficiency choices		Details
	Reference	Low material consumption	high material consumption	
**Clothing**				
Clothing materials	50%-50% cotton(knit-woven)	Silk	polypropylene	Different materials,cotton, wool and rubber, the cotton is arbitrary repleace for other materials cotton_woven, cotton_knit,silk,polypropylene,polyester,kenaf,jute
Amount of clothes	2,4 kg	_	4,8kg	Millward-Hopkins et al., (2020), arbitrary increase of 100% to account for cold regions
Frecuency of washing	77,86 kg/year	_	38,93 kg/year	Millward-Hopkins et al., (2020), arbitrary increase of 100% of washing to account warm regions
Efficiency Washing/drying	C	efficient(91% washing, 65% dryer)	D	Gooijer & Stamminger (2016), Efficient rates from A to D and an efficient case from Cullen et al. (2011)
Drying machine	yes	no	_	Include not using dryer machine to account for warm regions
**Education**				
Building type	standard,.RES0. Climate temperature (France)	efficient,.RES2.1. Climate temperature (France)	ZEB,.RES0. Climate temperature (France)	Data for the efficiency and building materials from the RECC model
Space required	10 m2/pupil	_	15m2/pupil	Reference from Millward-Hopkins et al., (2020), arbitrary 50% increase
Ilumination intensity	430lm/m2	215 lm/m2	_	Values from Cullen et al., (2011), reduction of 50% as not all the rooms need to be fully illuminated and that a proportion of the buildings are hallways that required 54 lm/ m2
Energy source heating	Natural gas	_	Electricty	
**Healtcare**				
Building type	standard,.RES0. Climate temperature (France)	Non-standard,.RES2.1.Warm temperature (India)	RES0_standard.Warm temperature (India)	Data for the efficiency and building materials from the RECC model
Space required per bed	180 m2	_	200 m2	Values from Millward-Hopkins et al., (2020)
Number beds	8 beds/1000 citizens	_	10 beds/1000 citizens	Values from Millward-Hopkins et al., (2020)
Ilumination intensity	430lm/m2	215 lm/m2	_	Values from Cullen et al., (2011), reduction of 50% as not all the rooms need to be fully illuminated and that a proportion of the buildings are hallways that required 54 lm/ m2
Energy source heating	Natural gas	_	Electricty	

Table S1.16. (continued)				
	Input parameters	lifestyle and efficiency choices		Details
	Reference	Low material consumption	high material consumption	
**Communication**				
Phone use intensity	Heavy(7,7 kWh/year)	Light(2,6 kWh/year)	_	Three intensity use heavy, light und average from Ercan et al., (2016)
Lapton use intensity	Average(62 kWh/year)	_	_	
Internet use intensity	standard(245 kWh/year)	25% more efficient	25% less efficient	Reference value from Ercan et al.,(2016) multiplied for 5 as suggested in Millward-Hopkins et al., (2020)
**Mobility**				
**pkm/(cap*year)**	8227	_	_	Based on Millward-Hopkins et al., (2020) formulations
Mobility scenario	RTS	_	2DS	Three scenarios RTS; 2DS and B2DS from the IEA (2017)
Transport modes technologies	Conventional gas and diesel vehicles	walking with conventional vehicles	Electric vehicles	A set of different transport technologes electro vehicles, bikes, scooters, bus, train, aircraft, with walking options
**Collective_services**				
Building type	Non-standard RES0 buildings (climate temperature)	Non-standard,.RES2.1 (climate temperature)	Non-standard,.RES0 (warm temperature)	Data for the efficiency and building materials from the RECC model
Space required	4,69m2	_	9,39m2	We use the half of the value from BPIE (2011) for Europe as reference, and the original value as upper bound. Education and Health buildings excluded to avoid doble accounting
Ilumination intensity	430lm/m2	215 lm/m2	_	Values from Cullen et al., (2011), reduction of 50% as not all the rooms need to be fully illuminated and that a proportion of the buildings are hallways that required 54 lm/ m2
Energy source heating	Natural gas	_	Electricty	

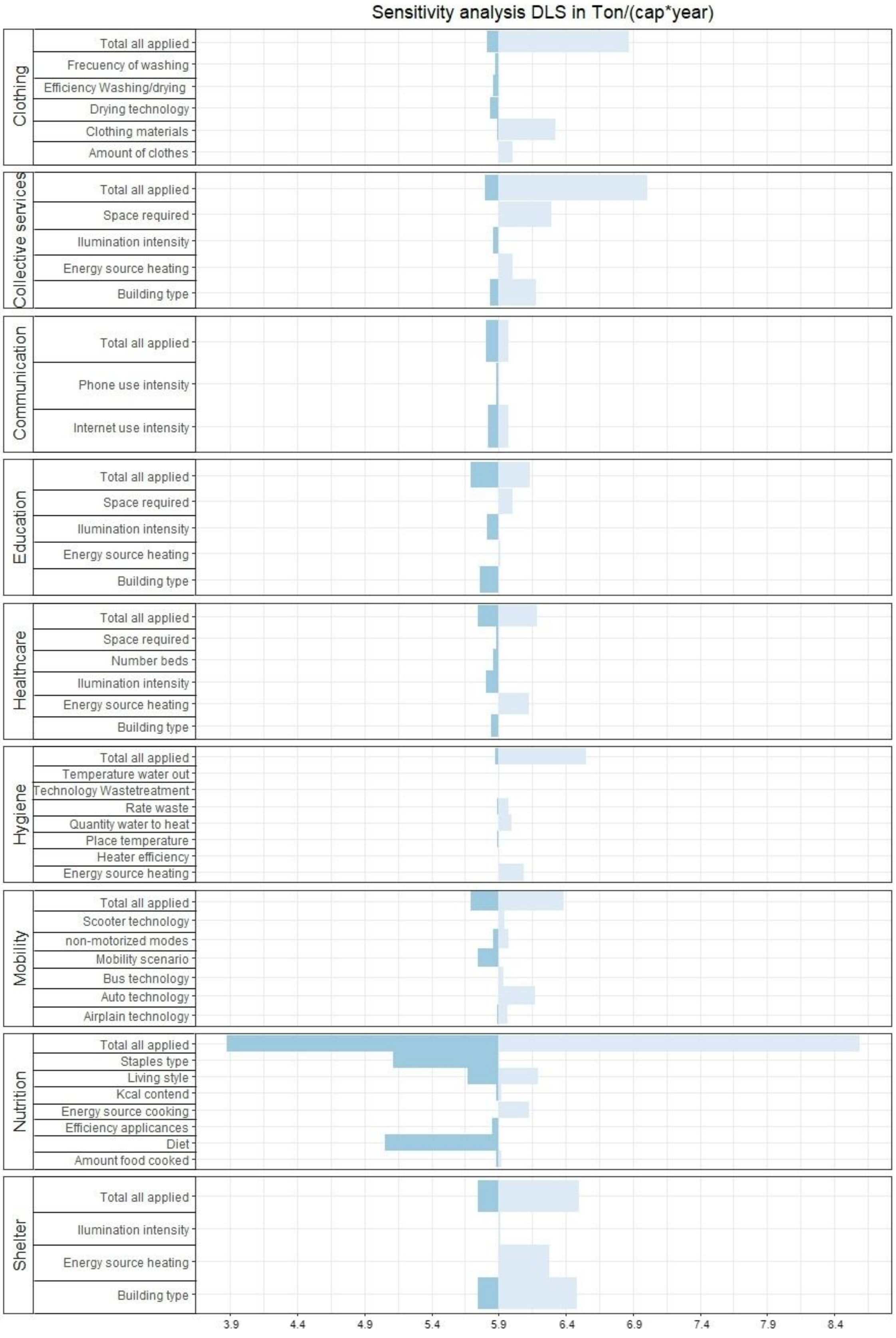

Figure S.1.2. Result sensitivity analysis, with parameter perturbations applied individually and then together sector-by-sector (named *all applied* in each case). The material footprint

for the reference scenario is 5.9 ton/cap*yr. Data for the figure are provided in the supplementary information S8.1.

4. References

(1) Haberl, H.; Wiedenhofer, D.; Erb, K. H.; Görg, C.; Krausmann, F. The Material Stock-Flow-Service Nexus: A New Approach for Tackling the Decoupling Conundrum. *Sustain.* **2017**, *9* (7). https://doi.org/10.3390/su9071049.

(2) Rao, N. D.; Min, J. Decent Living Standards: Material Prerequisites for Human Wellbeing. *Soc. Indic. Res.* **2018**, *138* (1), 225–244. https://doi.org/10.1007/s11205-017-1650-0.

(3) Millward-hopkins, J.; Steinberger, J. K.; Rao, N. D.; Oswald, Y. Providing Decent Living with Minimum Energy%: A Global Scenario. *Glob. Environ. Chang.* **2020**, *65* (August), 102168. https://doi.org/10.1016/j.gloenvcha.2020.102168.

(4) Heijungs, R.; Suh, S. *The Computational Structure of Life Cycle Assessment*; 2013.

(5) Grubler, A.; Wilson, C.; Bento, N.; Boza-Kiss, B.; Krey, V.; McCollum, D. L.; Rao, N. D.; Riahi, K.; Rogelj, J.; De Stercke, S.; Cullen, J.; Frank, S.; Fricko, O.; Guo, F.; Gidden, M.; Havlík, P.; Huppmann, D.; Kiesewetter, G.; Rafaj, P.; Schoepp, W.; Valin, H. A Low Energy Demand Scenario for Meeting the 1.5 °c Target and Sustainable Development Goals without Negative Emission Technologies. *Nat. Energy* **2018**, *3* (6), 515–527. https://doi.org/10.1038/s41560-018-0172-6.

(6) Pauliuk, S.; Heeren, N.; Berrill, P.; Fishman, T.; Nistad, A.; Tu, Q.; Wolfram, P.; Hertwich, E. Database of the ODYM-RECC v2.4 Model, Used for the GLOBAL Case Study on Material Efficiency and Climate Change Mitigation [Data Set]. Zenodo. 2021. https://doi.org/doi.org/10.5281/zenodo.4671644.

(7) Pauliuk, S.; Heeren, N.; Berrill, P.; Fishman, T.; Nistad, A.; Tu, Q.; Wolfram, P.; Hertwich, E. G. Global Scenarios of Resource and Emission Savings from Material Efficiency in Residential Buildings and Cars. *Nat. Commun.* **2021**, *12* (1). https://doi.org/10.1038/s41467-021-25300-4.

(8) Springmann, M.; Godfray, H. C. J.; Rayner, M.; Scarborough, P. Analysis and Valuation of the Health and Climate Change Cobenefits of Dietary Change. *Proc. Natl. Acad. Sci. U. S. A.* **2016**, *113* (15), 4146–4151. https://doi.org/10.1073/pnas.1523119113.

(9) Ecoinvent. *Impact Factors*. https://www.ecoinvent.org/ (accessed 2019-05-22).

(10) FAO. Energy-Smart Food for People and Climate. *Food and Agriculture Organization of the United Nations.* Rome 2011, p 78.

(11) Gleick, P. H. Basic Water Requirements for Human Activities: Meeting Basic Needs. *Water Int.* **1996**, *21* (2), 83–92. https://doi.org/10.1080/02508069608686494.

(12) Steinberger, J. K.; Friot, D.; Jolliet, O.; Erkman, S. A Spatially Explicit Life Cycle Inventory of the Global Textile Chain. *Int. J. Life Cycle Assess.* **2009**, *14* (5), 443–455. https://doi.org/10.1007/s11367-009-0078-4.

(13) Gooijer, H.; Stamminger, R. Water and Energy Consumption in Domestic Laundering Worldwide - A Review. *Tenside, Surfactants, Deterg.* **2016**, *53* (5), 402–409. https://doi.org/10.3139/113.110456.

(14) Cullen, J. M.; Allwood, J. M.; Borgstein, E. H. Reducing Energy Demand: What Are the Practical Limits? *Environ. Sci. Technol.* **2011**, *45* (4), 1711–1718. https://doi.org/10.1021/es102641n.

(15) Ercan, M.; Malmodin, J.; Bergmark, P.; Kimfalk, E.; Nilsson, E. Life Cycle Assessment of a Smartphone. In *4th International Conference on ICT for Sustainability*; 2016. https://doi.org/10.2991/ict4s-16.2016.15.

(16) Proske, M.; Clemm, C.; Richter, N. *LCA of the Fairphone 2*; Berlin, 2016. https://www.fairphone.com/wp-content/uploads/2016/11/Fairphone_2_LCA_Final_20161122.pdf.

(17) Deng, L.; Babbitt, C. W.; Williams, E. D. Economic-Balance Hybrid LCA Extended with Uncertainty Analysis: Case Study of a Laptop Computer. *J. Clean. Prod.* **2011**, *19* (11), 1198–1206. https://doi.org/10.1016/j.jclepro.2011.03.004.

(18) IEA. *Energy Technology Perspectives (ETP) - Catalysing Energy Technology Transformations, 2017.*; Paris, 2017. https://www.iea.org/reports/etp-model-2017.

(19) BPIE. *Europe´s Buildings under the Microscope. A Country-by-Country Review of the Energy Performance of Buildings*; Buildings Performance Institute Europe, 2011. http://www.bpie.eu/publication/europes-buildings-under-the-microscope/.
